# Programming of macrophages by UV-irradiated apoptotic cancer cells inhibits cancer progression and lung metastasis

**DOI:** 10.1038/s41423-019-0209-1

**Published:** 2019-03-06

**Authors:** Yong-Bae Kim, Young-Ho Ahn, Ji-Hae Jung, Ye-Ji Lee, Jin-Hwa Lee, Jihee Lee Kang

**Affiliations:** 1 Tissue Injury Defense Research Center, College of Medicine, Ewha Womans University, Seoul, 07804 Korea; 2 Department of Molecular Medicine, College of Medicine, Ewha Womans University, Seoul, 07804 Korea; 3 Department of Physiology, College of Medicine, Ewha Womans University, Seoul, 07804 Korea; 4 Department of Internal Medicine, College of Medicine, Ewha Womans University, Seoul, 07804 Korea

**Keywords:** Apoptotic cell clearance, EMT, Metastasis, Exosomal PTEN, PPARγ ligands, Phagocytes, Cancer microenvironment, Cancer microenvironment

## Abstract

Apoptotic cell clearance by phagocytes is essential in tissue homeostasis. We demonstrated that conditioned medium (CM) from macrophages exposed to apoptotic cancer cells inhibits the TGFβ1-induced epithelial–mesenchymal transition (EMT), migration, and invasion of cancer cells. Apoptotic 344SQ (ApoSQ) cell-induced PPARγ activity in macrophages increased the levels of PTEN, which was secreted in exosomes. Exosomal PTEN was taken up by recipient lung cancer cells. ApoSQ-exposed CM from PTEN knockdown cells failed to enhance PTEN in 344SQ cells, restore cellular polarity, or exert anti-EMT and anti-invasive effects. The CM that was deficient in PPARγ ligands, including 15-HETE, lipoxin A4, and 15d-PGJ_2_, could not reverse the suppression of PPARγ activity or the PTEN increase in 344SQ cells and consequently failed to prevent the EMT process. Moreover, a single injection of ApoSQ cells inhibited lung metastasis in syngeneic immunocompetent mice with enhanced PPARγ/PTEN signaling both in tumor-associated macrophages and in tumor cells. PPARγ antagonist GW9662 reversed the signaling by PPARγ/PTEN; the reduction in EMT-activating transcription factors, such as *Snai1 and Zeb1*; and the antimetastatic effect of the ApoSQ injection. Thus, the injection of apoptotic lung cancer cells may offer a new strategy for the prevention of lung metastasis.

## Introduction

Metastasis is a complex multistep process of cancer cell dissemination and is extremely difficult to treat. The outcomes of cancer patients with metastatic disease have not improved over the past 30 years, despite the development of targeted therapies.^1^ In one working hypothesis, metastasis is initiated by tumor cells that undergo the epithelial-to-mesenchymal transition (EMT) in response to extracellular signals, leading to loss of polarity, detachment from neighboring cells, increased motility, invasion into the surrounding matrix, and resistance to standard cytotoxic chemotherapy.^2,3^

*PTEN* (phosphatase and tensin homolog on chromosome ten), a powerful and multifaceted suppressor, is mutated in multiple types of cancer and has both phosphatase-dependent and phosphatase-independent roles.^4^ PTEN antagonizes phosphoinositide 3-kinase (PI3K) signaling and thereby affects several cellular processes, including growth, proliferation, and survival.^5,6^ A number of clinical studies have demonstrated that PTEN suppression or loss in advanced-stage disease contributes to the EMT induction associated with tumor invasion and metastasis.^7,8^ PTEN knockdown in human colon cancer cells or prostate cancer cells leads to EMT induction, associated with invasion and metastasis.^9^ In mice, PTEN loss results in neoplastic growth, in both tumors and the tumor microenvironment.^10,11^ Peroxisome proliferator-activated receptor gamma (PPARγ) is a potential PTEN transcription factor; its activation through ligands increases functional PTEN protein expression in various cancer cell lines, subsequently inhibiting Akt phosphorylation and cellular growth.^12–14^ Several in vivo studies have demonstrated that genetic alterations in PPARγ can promote tumor progression.^15,16^ These studies suggest the importance of PPARγ/PTEN signaling in cancer prevention.

Cell death can be classified according to its morphological appearance, which may be apoptotic or necrotic.^17^ Apoptosis is a mechanism for the removal of unwanted or damaged cells in the maintenance of normal tissue homeostasis. Apoptosis is usually associated with the retention of plasma membrane integrity, the condensation and degradation of cytoskeletal and nuclear proteins, and the formation of apoptotic bodies. The morphological features of apoptosis result from the activation of caspases by either death receptor ligation or the release of apoptotic mediators from the mitochondria.^18,19^ Apoptotic death can be triggered by a wide variety of different stimuli, including TNF, TGF-β1, genotoxic factors, oxidants, ultraviolet irradiation, and gamma irradiation.^20^ In contrast, necrosis has been described as a consequence of extreme physicochemical stress, resulting in widespread destruction of the cell, including the nucleus and cell membrane.^21^ One distinction between apoptosis and necrosis is that apoptosis usually elicits anti-inflammatory responses, while necrosis promotes inflammation.^22,23^

Apoptotic cell clearance by tissue macrophages and nonprofessional phagocytes is essential for tissue homeostasis, immunity, and inflammation resolution. High levels of cell death can occur within the tumor environment, and clearance mechanisms for dying tumor cells can profoundly influence tumor-specific immunity. Recognition of phosphatidylserine exposed on the surfaces of apoptotic cells has been shown to stimulate their uptake and removal by phagocytes, as well as the production of immunosuppressive cytokines, such as TGF‐β, IL‐10, and PGE_2_.^24^ Furthermore, recent data indicate that apoptotic cell clearance results in the release of growth factors, such as HGF and VEGF, used for epithelial and endothelial maintenance.^25,26^ Thus, the engulfment of apoptotic cells coupled with cytokine modulation aimed at immune suppression ensures that apoptotic cell death does not induce inflammation or tissue damage. However, cytokines involved in wound healing and immune suppression are notorious for their roles in the tumor microenvironment, increasing the EMT process of tumor cells and promoting the evasion of antitumor immunity.^27^ In particular, recent studies have provided evidence that the TGF-β1-induced EMT of many epithelial cancer cells may contribute to fibrotic diseases and cancer progression.^28,29^ However, it was demonstrated that the in vitro and in vivo exposure of macrophages to apoptotic cells inhibits TGF-β1 or bleomycin-induced EMT in lung alveolar epithelial cells.^30^ Whether the efferocytosis of apoptotic cells affects the multistep process of cancer cell dissemination, leading to cancer metastasis, has not been studied thus far. Here, using in vitro 2D- and 3D-culture systems, we investigate whether the interaction between macrophages and dying lung cancer cells inhibits EMT in lung epithelial cancer cells and decreases cancer cell migration and invasiveness. We demonstrate that PTEN secretion in exosomes and the PPARγ ligands from macrophages exposed to apoptotic lung cancer cells block the multistep metastatic process. Furthermore, we provide in vivo evidence that the subcutaneous injection of apoptotic lung cancer cells decreases the number of visible lung metastases of the primary subcutaneous tumor via PPARγ/PTEN signaling.

## Results

### Interaction between macrophages and UV-irradiated apoptotic lung cancer cells inhibits EMT in cancer cells

**Fig. 1:**
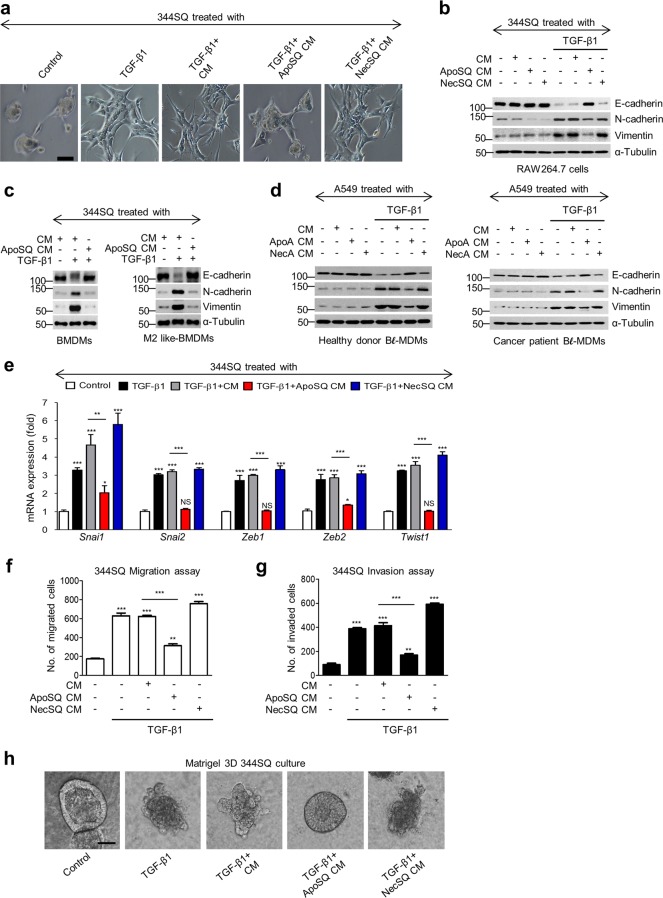
The interaction between macrophages and UV-irradiated apoptotic lung cancer cells inhibits EMT, migration, and invasion in cancer cells. **a**, **b**, **e**–**h** RAW cells were stimulated with apoptotic (ApoSQ) or necrotic (NecSQ) 344SQ cells for 24 h. **c** Mouse bone marrow-derived macrophages (BMDMs) (*left*) or M2-like BMDMs (*right*) were stimulated with ApoSQ or NecSQ. **d** Blood monocyte-derived macrophages (MDMs) from healthy donors (*left*) or lung cancer patients (*right*) were stimulated with apoptotic (ApoA) or necrotic (NecA) A549 cells for 24 h. Conditioned medium (CM) was added to 344SQ cells, shown in (**a**–**c**, **e**–**h)**, or A549 cells, shown in (**d**), with or without TGF-β1 (10 ng/ml) for 48 h. **a** Morphological changes in cells were examined by phase-contrast microscopy. **b**–**d** Immunoblot analysis of indicated proteins in whole-cell lysates. **e** Gene expression in 344SQ cell samples was measured by real-time PCR. The quantification of migrated cells in Boyden chambers is shown in (**f**), and that of invaded cells in Boyden chambers is shown in (**g**). **h** 344SQ cells were cultured for 7 days in Matrigel before exposure to CM with TGF-β1 (10 ng/ml) for 72 h and photographed by phase-contrast microcopy. *P < 0.05, **P < 0.01, and ***P < 0.001. Data are from one experiment representative of three independent experiments, as shown in (**a**–**c**, **h**); of three donors, as shown in (**d**); with similar results; or from three independent experiments (mean ± s.e.m. in (**e**–**g**). Scale bars: 100 μm in (**a**, **h**)

To determine whether the interaction between macrophages and apoptotic lung epithelial cancer cells inhibits EMT progression, 344SQ murine lung adenocarcinoma cells were treated with conditioned medium (CM) from RAW cells exposed to either UV-irradiated apoptotic 344SQ (ApoSQ-exposed CM) or necrotic 344SQ cells (NecSQ-exposed CM), along with TGF-β1. ApoSQ-exposed CM inhibited TGF-β1-induced EMT, based on morphological cellular alterations (Fig. 1a), and the EMT marker mRNA (Supplementary Fig. S1a) and protein (Fig. 1b) expression profiles. In contrast, NecSQ-exposed CM did not exhibit anti-EMT effects. Immunofluorescence using E-cadherin (green) and vimentin (red) monoclonal antibodies was performed to validate the changes in EMT marker proteins. As indicated by the western blotting data, the TGF-β1-induced decrease in E-cadherin expression and increase in vimentin expression in 344SQ cells were reversed by the ApoSQ-exposed CM, but not by the NecSQ-exposed CM (Supplementary Fig. S1b). In addition, the ApoSQ or NecSQ medium alone did not affect the TGF-β1-induced changes in EMT marker mRNA (Supplementary Fig. S2a–c) or protein expression in 344SQ cells (Supplementary Fig. S2d). Anti-EMT effects were also not observed with another important control, in which the ApoSQ or NecSQ medium was used to culture RAW cells (Supplementary Fig. S2e–h), suggesting that a factor released by apoptotic cancer cells does not allow macrophages to develop a similar ability to that observed in coculture.

We confirmed the anti-EMT effects of various types of apoptotic cancer cell-exposed CM in the human non-small-cell lung cancer (NSCLC) cell line A549 (Supplementary Fig. S3a–c) and other human cancer cell lines of the breast (MDA-MB-231), colon (COLO320HSR), and prostate (PC3) (Supplementary Fig. S3d–f). In addition to CM from RAW cells, CM from ApoSQ-exposed primary mouse bone marrow-derived macrophages (BMDMs), or from those with the IL-4-derived M2 phenotype, substantially inhibited TGF-β1-induced changes in EMT markers in 344SQ cells (Fig. 1c). CM from blood monocyte-derived macrophages (MDMs) from healthy humans and NSCLC (adenocarcinoma) patients exposed to apoptotic A549 cells (ApoA) had anti-EMT effects (Fig. 1d; Supplementary Fig. S3g and h).

However, neither CM from nor direct exposure to epithelial cancer cells inhibited TGF-β1-induced EMT marker changes (Supplementary Fig. S4a–d), indicating that cancer cell EMT inhibition requires bioactive mediators that are secreted by professional phagocytes, such as macrophages, which are functionally altered by apoptotic cancer cell stimulation.

TGF-β-induced EMT is achieved through the well-orchestrated actions of the Snai, ZEB, and Basic helix–loop–helix transcription factor families.^31^ We observed that ApoSQ- or apoptotic A549 (ApoA)-exposed CM markedly inhibited TGF-β1-induced *Snai1/2*, *Zeb1/2*, and *Twist1* mRNA expression, whereas control, NecSQ-exposed, or necrotic A549 (NecA)-exposed CM did not (Fig. 1e; Supplementary Fig. S5a). Intracellular signaling experiments (Supplementary Fig. S5b) showed that Smad-dependent TGF-β signaling and the ERK pathway were not affected (Supplementary Fig. S5c–e). However, ApoSQ-exposed CM partially blocked Smad-independent TGF-β signaling, including the p38 MAP kinase and Akt pathways, in 344SQ cells (Supplementary Fig. S5f and g).

### Interaction between macrophages and apoptotic cancer cells inhibits lung cancer cell migration and invasion

Acquisition of the mesenchymal phenotype by malignant cancer cells is associated with decreased cell–cell adhesion and increased migratory and invasive properties, which are crucial for metastasis.^32^ Our data show that ApoSQ- or ApoA-exposed CM prevented TGF-β1-induced cancer cell migration and invasion (Fig. 1f, g; Supplementary Fig. S6a and b), whereas control, NecSQ-exposed, and NecA-exposed CM did not. Moreover, 3D Matrigel culture confirmed the anti-invasive effect of ApoSQ-exposed CM, which caused 344SQ cells to recover their lost polarity and acinus-like colonies induced by TGF-β1 (Fig. 1h).

### PPARγ-dependent PTEN secretion in exosomes from macrophages and entry into recipient cancer cells

**Fig. 2:**
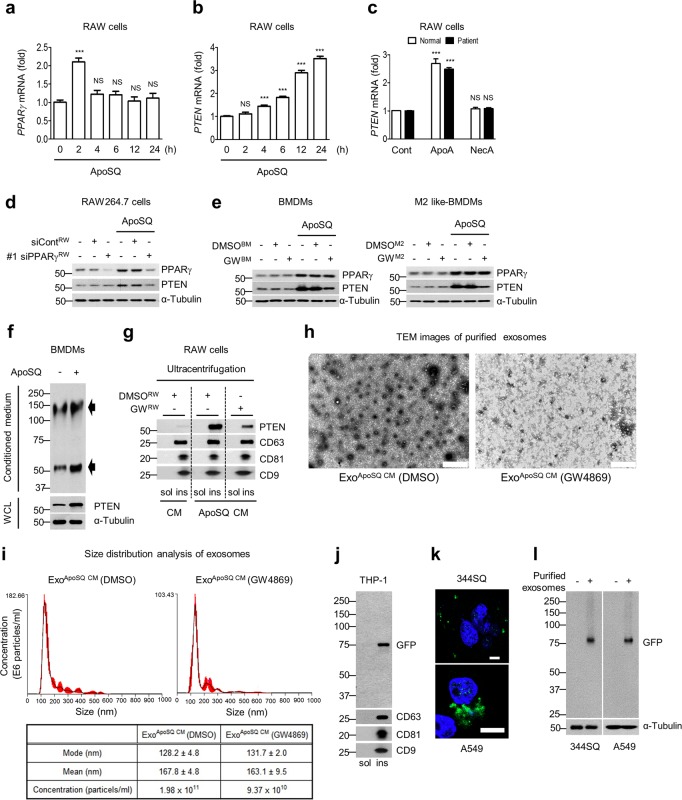
PPARγ-dependent PTEN secretion in exosomes and PTEN uptake by recipient cells. qPCR analysis of *PPARγ* and *PTEN* mRNA in RAW cells exposed to apoptotic 344SQ cells (ApoSQ) for the indicated times shown in (**a**, **b**) or *PTEN* mRNA in blood MDMs from healthy donors or lung cancer patients exposed to apoptotic (ApoA) or necrotic (NecA) A549 cells for 24 h, as shown in (**c**). **d** Immunoblot analysis of indicated proteins in RAW cells transfected with PPARγ siRNA before ApoSQ stimulation for 24 h. **e** Immunoblot analysis of indicated proteins in BMDMs (*left*) or M2-like BMDMs (*right*) pretreated with GW9662 (10 μM) for 1 h before stimulation with ApoSQ for 24 h. **f** Immunoblot analysis of PTEN with whole-cell lysates from mouse BMDMs stimulated with ApoSQ (WCL) and of the secretion levels of PTEN using affinity pull-down in conditioned medium (CM). PTEN immunoprecipitates were separated by SDS-PAGE in nonreducing conditions. The arrows indicate the immunoglobulin heavy/light chain complex (up) and PTEN (down). **g** CM from RAW cells pretreated with 10 μM of GW9662 before ApoSQ stimulation for 24 h was fractionated by ultracentrifugation, and the soluble (sol) and insoluble (ins) fractions were immunoblotted with antibodies against PTEN, CD63, CD81, or CD9. **h** TEM images of exosomes isolated from the CM of ApoSQ-stimulated RAW cells pretreated with or without 20 μM GW4869. Scale bars: 20 μm. **i** Size distribution analysis of exosomes from the CM of ApoSQ-stimulated RAW cells pretreated with 20 μM GW4869 or vehicle (2% DMSO in saline). The horizontal axis represents particle size (nm), and the vertical axis represents particle concentration (×10^6^ particles/ml). The red bars represent the s.e.m. The values represent the mode or mean size ± s.e.m. from three independent experiments. **j** Immunoblot analysis of GFP-PTEN, CD63, CD81, and CD9 in the soluble and insoluble fractions after ultracentrifugation of CM from a human macrophage cell line (hMϕ) overexpressing GFP-PTEN exposed to ApoA. **k** Direct fluorescence of 344SQ and A549 cells 24 h after treatment with harvested exosomes containing GFP-PTEN using confocal microcopy. Scale bars: 20 μm. **l** Lysates from 344SQ and A549 cells after incubation with harvested exosomes from hMϕ overexpressing GFP-PTEN were subjected to western blotting analysis. NS not significant; ^**^P < 0.01 and ^***^P < 0.001. Data are from three independent experiments (mean ± s.e.m. in (**a**, **b**, **i**) below tables), three donors (mean ± s.e.m. in (**c**)), or one experiment representative of three independent experiments with similar results, as shown in (**d**–**l**)

PPARγ expression and activity are enhanced in macrophages exposed to apoptotic cells in vitro and in vivo.^33,34^ We hypothesized that PPARγ-dependent PTEN production in macrophages exposed to apoptotic lung cancer cells plays a crucial role in the anti-EMT effect. To prove this, we examined PPARγ expression and activity and PTEN production in ApoSQ-exposed RAW cells. PPARγ mRNA and protein expression and its activity were markedly enhanced (Fig. 2a; Supplementary Fig. S7a–d). *PPARγ* mRNA expression was enhanced in an apoptotic cell number-dependent manner (Supplementary Fig. S7a). In parallel, PTEN mRNA and protein levels were markedly increased (Fig. 2b; Supplementary Fig. S7b and c). *PTEN* mRNA expression in blood MDMs from healthy humans or lung cancer patients was also enhanced by ApoA exposure (Fig. 2c). Neither NecSQ nor NecA exerted these effects. Inhibition of PPARγ activity by GW9662 (Supplementary Fig. S7d–f) or PPARγ knockdown with two siRNAs (Fig. 2d; Supplementary Fig. S7g) reversed the PTEN mRNA and protein increases in ApoSQ-exposed RAW cells. Similar PPARγ-dependent PTEN induction was shown in naive BMDMs and M2 BMDMs exposed to ApoSQ (Fig. 2e). Notably, treatment of RAW 264.7 cells, BMDMs and M2 BMDMs with GW9662 had no effects on PPARγ mRNA or protein expression. These data suggest that this important protein is transcriptionally upregulated by enhanced PPARγ expression and activation in macrophages, upon stimulation with apoptotic cancer cells.

Interestingly, GW9662 treatment of RAW cells significantly reversed the anti-EMT and anti-invasive effects of ApoSQ-exposed CM (Supplementary Fig. S8a–d). PPARγ-dependent PTEN production in this experimental context might be a candidate for the acquisition of these anti-EMT and anti-invasive effects by the CM, if PTEN can be secreted in exosomes and if the secreted PTEN is internalized by recipient cells, with consequent functional activity. To confirm this assumption, we first examined whether the enhanced PTEN protein levels could be secreted in exosomes from macrophages. We observed strong PTEN expression in ApoSQ-exposed CM (Fig. 2f). Moreover, exosomes were isolated by sequential ultracentrifugation and used for western blotting analysis using anti-PTEN and exosomal marker antibodies.^35^ Importantly, PTEN was recovered in the insoluble fraction with exosomal markers, such as CD63, CD81, and CD9, confirming the presence of PTEN in ApoSQ-exposed CM, whereas GW9662 treatment reduced PTEN abundance (Fig. 2g). In addition, to reveal the role of exosomes for delivery, we pretreated RAW cells with 20 μM GW4869, an inhibitor of exosome biogenesis/release, followed by treatment with ApoSQ. Exosomes secreted from macrophages were isolated by sequential ultracentrifugation, morphologically analyzed by transmission electron microscopy (TEM), and characterized by nanoparticle tracking analysis (NTA) using NanoSight, given their small sizes (80–160 nm).^36,37^ TEM revealed that the isolates contained nanosized vesicles and that the number of vesicles in a unit area appeared to be decreased by treatment with GW4869 (Fig. 2h). While the size distribution of the exosomes from macrophages with or without GW4869 did not change, the concentration of exosomes present was decreased upon GW4869 treatment (Fig. 2i).

To ascertain the entry of PTEN-bearing exosomes secreted from macrophages into the recipient cancer cells, CM from human macrophages overexpressing green fluorescent protein (GFP)-PTEN exposed to ApoA was subjected to sequential ultracentrifugation for the harvesting of exosomes. We confirmed the presence of exosomal PTEN in the insoluble fraction through western blotting analysis using GFP, CD63, CD81, and CD9 antibodies (Fig. 2j). After treating the 344SQ and A549 cells with the harvested exosomes for 24 h, we examined GFP fluorescence in the cells using confocal microscopy (Fig. 2k), and the results indicated the uptake of exosomal GFP-PTEN. This observation was confirmed using western blotting with anti-GFP antibodies (Fig. 2l).

### PTEN alters signaling, promotes cellular polarity, and inhibits EMT upon entry into 344SQ cells

**Fig. 3:**
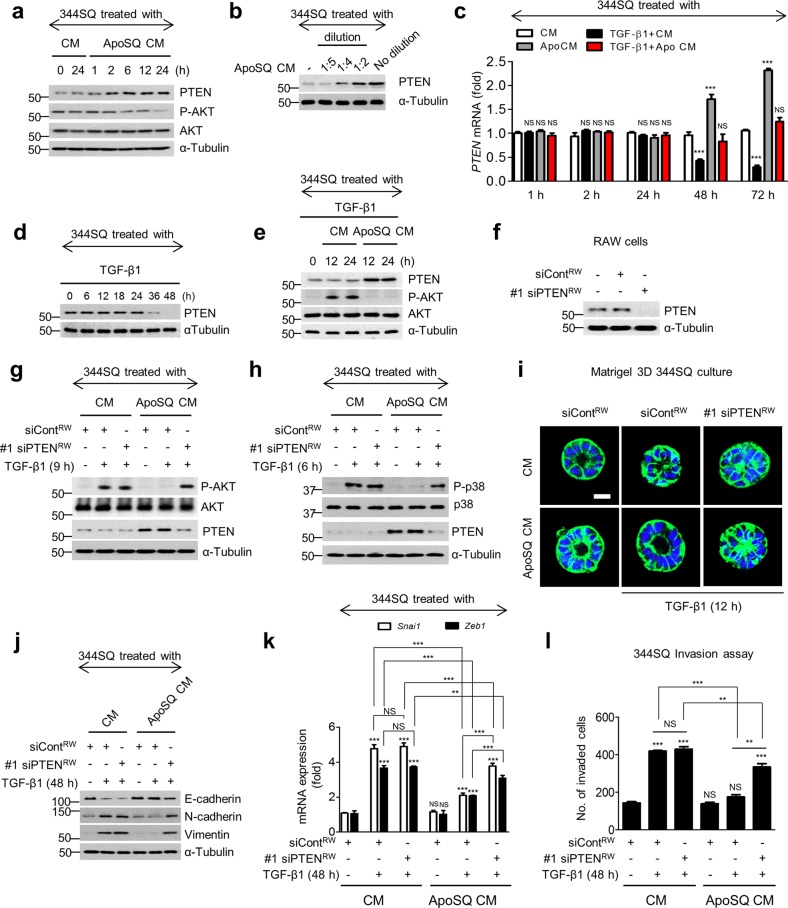
Internalized PTEN alters signaling, retains polarity, and inhibits the EMT and invasion of 344SQ cells. **a** Immunoblot analysis of indicated proteins in 344SQ cell lysates after incubation with CM for the indicated times. **b** Immunoblot analysis of PTEN in 344SQ cells after incubation with ApoSQ-exposed CM at various dilutions (from 1/5 to 1) with control CM for 12 h. The qPCR analysis of *PTEN* mRNA is shown in (**c**). The immunoblot analysis of indicated proteins in 344SQ cells after the addition of TGF-β1 (10 ng/ml) is shown in (**d**) and that after the addition of CM is shown in **(e)**. **f**–**l** RAW cells were transfected with PTEN siRNA (#1 siPTEN) before ApoSQ cell stimulation for 24 h. **f** Immunoblot analysis of PTEN in RAW cells. **g**–**l** CM was added to 344SQ cells with or without TGF-β1 (10 ng/ml) for the indicated times. **g**, **h**, **j** Immunoblot analysis of indicated proteins in 344SQ cells. **i** Confocal microscopic images of 3D acini at 12 h after treatment with TGF-β1 (10 ng/ml). Normal acini of 344SQ cells were grown in 3D Matrigel containing CM and stained with anti-β-catenin (green) and DAPI. Scale bars: 20 μm. **k** qPCR analysis of *Snai1* and *Zeb1* mRNAs in 344SQ cells. **l** The numbers of invaded cells were analyzed to assess their invasive ability using Matrigel-coated Transwells. NS not significant; ***P *<* 0.001. Data are from three independent experiments (mean ± s.e.m. in (**c**, **k**, **l**)) or one experiment representative of three independent experiments with similar results, as shown in (**a**, **b**, **d**–**j**)

Following treatment with ApoSQ-exposed CM, PTEN protein levels in recipient 344SQ cells increased immediately and remained increased up to 24 h, and basal Akt phosphorylation decreased reciprocally over 6 h (Fig. 3a). Moreover, PTEN expression was directly proportional to the concentration of ApoSQ-exposed CM supernatant (Fig. 3b), although the mRNA abundance of PTEN was not affected until 24 h after ApoSQ-exposed CM treatment in the absence or presence of TGF-β1 (Fig. 3c). TGF-β1 itself did not affect the basal PTEN protein abundance for 24 h, but thereafter caused it to decrease in 344SQ cells (Fig. 3d). In contrast to PTEN abundance, TGF-β1-induced Akt phosphorylation decreased up to 24 h after ApoSQ-exposed CM treatment (Fig. 3e). However, PTEN enhancement and the consequent reductions in Akt phosphorylation and p38 MAP kinase activity were not seen with ApoSQ-exposed CM from RAW cells transfected with two PTEN siRNAs (Fig. 3f–h; Supplementary Fig. S9a–c). These data suggest that PTEN is functionally active toward altering basal Akt signaling and TGF-β1-induced non-Smad signaling.

Loss of PTEN function prevents normal apical surface and lumen development in 3D-culture.^38^ Thus, to evaluate the role of PTEN in cell polarity, 344SQ cells grown in 3D Matrigel were treated with CM from RAW cells transfected with two PTEN siRNAs, stained with anti-β-catenin (green), and examined by confocal microscopy. Treatment with ApoSQ-exposed CM from control siRNA-transfected macrophages prevented TGF-β1-induced interference, with the formation of polarized acinar structures by 344SQ cells relatively early in the exposure process (12 h after TGF-β1 treatment) (Fig. 3i; Supplementary Fig. S9d). ApoSQ-exposed CM from PTEN knockdown macrophages did not exert this inhibitory effect.

Next, we examined whether PTEN contributes to the late-phase anti-EMT and anti-invasive effects of ApoSQ-exposed CM. PTEN knockdown in RAW cells reversed the inhibition of the TGF-β1-induced changes in EMT marker expression at the protein level; the increases in mRNA expression of Zeb1/2, Snail1/2, and Twist1; and the number of invading 344SQ cells relatively late in the process of exposure (48 h after TGF-β1 treatment) to the ApoSQ-exposed CM (Fig. 3j–l; Supplementary Fig. S9e–g). These data indicate that macrophage-derived PTEN mediates the anti-EMT and anti-invasive effects of ApoSQ-exposed CM during the late phase in TGF-β1-stimulated 344SQ cells.

### Purified exosomes from macrophages downregulate EMT, Akt/p38 signal cascades, and cancer cell invasion

**Fig. 4:**
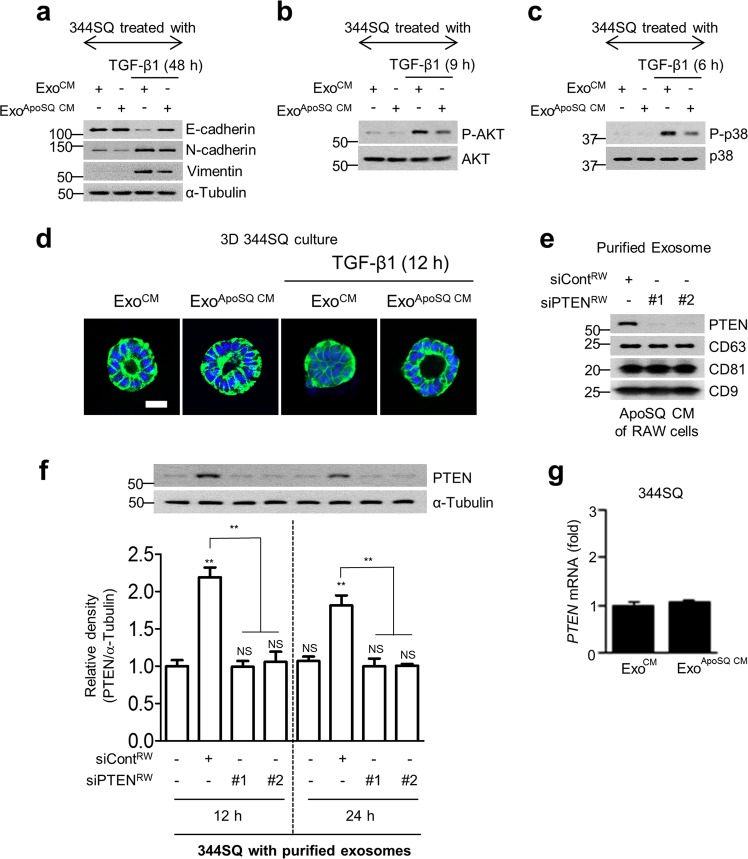
Downregulation of EMT, the Akt/p38 signaling cascades, and cancer cell invasion by purified exosomes and the exosomal PTEN level in recipient cancer cells. 344SQ cells were treated with exosomes isolated from RAW conditioned medium with (Exo^ApoSQ CM^) or without (Exo^CM^) apoptotic 344SQ cells, in the presence of TGF-β1 (10 ng/ml) for the indicated times, as shown in (**a**–**d**). **a**–**c** Immunoblot analysis of indicated proteins in 344SQ cell lysates after incubation with exosomes. **d** Confocal microscopic images of 3D acini were taken 10 days after cell seeding. The normal acini of 344SQ cells were grown in 3D Matrigel containing exosomes and stained with anti-β-catenin (green) and DAPI. Scale bars: 20 μm. 344SQ cells were treated with purified exosomes from the culture medium of apoptotic cancer cell-stimulated RAW 264.7 cells transfected with or without two siRNAs for PTEN (#1 and #2 siPTEN) for the indicated times, as shown in (**e**, **f**). The immunoblot analysis of indicated proteins in purified exosomes is shown in (**e**), that in 344SQ cell lysates after treatment with purified exosomes is shown in (**f**). **g** qPCR analysis of *PTEN* mRNA in 344SQ cells 12 h after treatment with exosomes isolated from RAW CM with (Exo^ApoSQ CM^) or without (Exo^CM^) apoptotic 344SQ cells. NS not significant; **P < 0.01. Data are from one experiment representative of three independent experiments with similar results are shown in (**a**–**e**, **f** upper panel), or data from three independent experiments (mean ± s.e.m.) are shown in (**f** lower panel) and (**g**)

To determine the effects of secreted exosomes from macrophages on EMT, the Akt/p38 signal cascades, and cancer cell invasion, purified exosomes from ApoSQ-exposed CM were added to 344SQ cells with TGF-β1. Treatment with purified exosomes from ApoSQ-exposed CM inhibited TGF-β1-induced changes in EMT markers and the phosphorylation of Akt and p38 MAP kinase in 344SQ cells (Fig. 4a–c). In addition, treatment with purified exosomes reversed TGF-β1-induced inhibition of the formation of polarized acinar structures by 344SQ cells in 3D Matrigel culture, indicating an anti-invasive effect (Fig. 4d).

Next, to exclude the possibility that PTEN expression in recipient cancer cells could be stimulated by certain molecules in exosomes from macrophages, we examined PTEN protein levels in recipient cells after treatment with purified exosomes from the CM of ApoSQ-stimulated macrophages transfected with two PTEN siRNAs. PTEN levels in recipient 344SQ cells increased at 12 and 24 h following treatment with purified exosomes from control siRNA-transfected macrophages (Fig. 4e, f). However, PTEN expression was not enhanced by treatment with purified exosomes from PTEN knockdown macrophages. These data suggest that enhanced PTEN expression in recipient 344SQ cells originates from exosomal PTEN secreted from macrophages, but is not stimulated by certain molecules in exosomes. We further examined PTEN mRNA expression at 12 h after treatment with purified exosomes from ApoSQ CM or CM. The PTEN mRNA abundance in the recipient 344SQ cells was not affected at 12 h of treatment with purified exosomes (Fig. 4g). These data support the concept that the enhanced PTEN protein level in recipient 344SQ cells is not affected in the early phase by PTEN mRNA transcription stimulated by certain molecules in exosomes.

### Secreted PPARγ ligands from macrophages mediate anti-EMT effects in 344SQ cells via PPARγ/PTEN signaling

**Fig. 5:**
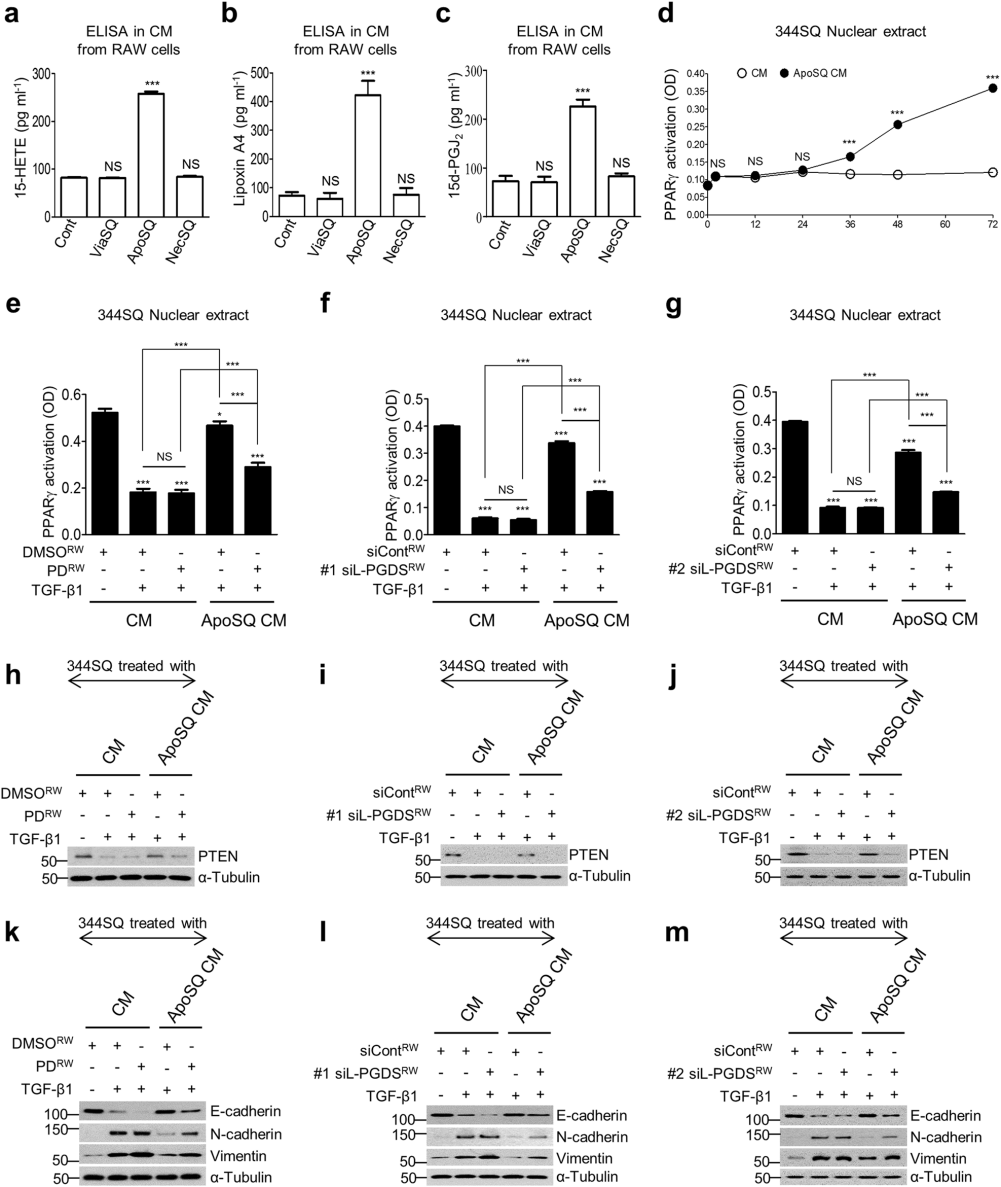
Macrophage secretion of 15-HETE, lipoxin A4, and 15d-PGJ_2_ mediates anti-EMT effects. **a**–**c** ELISA of 15-HETE, lipoxin A4 and 15d-PGJ_2_ in conditioned medium (CM) from RAW cells alone or from cocultures of RAW/viable 344SQ cells (ViaSQ), RAW/apoptotic 344SQ cells (ApoSQ), or RAW/necrotic 344SQ cells (NecSQ). **d** Time course of PPARγ activation in 344SQ cells with CM or ApoSQ CM at the indicated times. RAW cells were pretreated with 10 μM PD146176 (PD) for 1 h, as shown in (**e**, **h**, **k**), or transfected with two siRNAs against lipocalin-type prostaglandin D synthase (#1 and #2 siL-PGDS) for 48 h, as shown in (**f**, **g**, **i**, **j**, **l**, **m**) before stimulation with ApoSQ cells for 24 h. **e**–**m** CM was added to 344SQ cells with TGF-β1 (10 ng/ml) for 48 h. PPARγ activation is shown in (**e**–**g**), and the immunoblot analysis of indicated protein expression in 344SQ cells is shown in (**h**–**m**). NS not significant; ^*^P < 0.05 and ***P < 0.001. Data are from three independent experiments (mean ± s.e.m.) are shown in (**a**–**g**), and data from one experiment representative of three independent experiments with similar results are shown in (**h**–**m**)

Apoptotic cells stimulate the production of the identifiedPPARγ ligands, such as 15-lipoxygenase-dependent 15-hydroxyeicosatetraenoic acid (HETE), lipoxin A4, and PGD_2_ synthase-dependent 15d-deoxy-Δ12,14-prostaglandin J_2_ (PGJ_2_).^39,40^ Consistently, we observed enhanced production of these PPARγ ligands in ApoSQ-exposed CM (Fig. 5a–c). However, viable or necrotic 344SQ cells did not show these effects. PPARγ activity gradually increased in 344SQ cells over 36–72 h after treatment with ApoSQ-exposed CM in the absence of TGF-β1 (Fig. 5d). Consistent with previous findings, the *PPARγ* mRNA abundance (Supplementary Fig. S10a–c) and activity (Fig. 5e–g) were suppressed upon TGF-β1 stimulation, as were the PTEN mRNA (Fig. 3c; Supplementary Fig. S10d–f) and protein levels at 48 h (Fig. 5h–j).^41^ However, ApoSQ-exposed CM treatment reversed these reductions. To confirm that the PPARγ activity increase was mediated by secreted PPARγ ligands, RAW cells were pretreated with the 15-lipoxygenase inhibitor PD146176 or transfected with two lipocalin-type PGD synthase (L-PGDS) siRNAs before ApoSQ treatment. Under these conditions or with L-PGDS protein knockdown (Supplementary Fig. S11a and b), the production of 15-HETE, lipoxin A4, PGD_2_, and 15d-PGJ_2_ was substantially reduced (Supplementary Fig. S11c–h). Notably, treatment with this CM, deficient of PPARγ ligands, could not effectively reverse the TGF-β1-induced suppression of *PPARγ* mRNA (Supplementary Fig. S10a–c) or activity (Fig. 5e–g) or the effects on the PTEN mRNA (Supplementary Fig. S10d–f) and protein levels (Fig. 5h–j) 48 h after TGF-β1 treatment. Consequently, late EMT processes were not effectively prevented (Fig. 5k–m; Supplementary Fig. S11i–k). These data suggest that the macrophage secretion of these ligands mediates anti-EMT effects through enhanced PPARγ/PTEN signaling in recipient 344SQ cells.

Additionally, we examined whether PTEN in macrophages affects the production of PPARγ ligands, using PTEN siRNA. RAW cells were transfected with PTEN siRNA, before ApoSQ treatment. With PTEN protein knockdown (Fig. 3f), the production of 15-HETE, lipoxin A4, and 15d-PGJ2 was not reduced (Supplementary Fig. S12a–c). These data suggest that apoptotic cancer cell-induced PTEN may not affect the pathways generating these PPARγ ligands from membrane phospholipids. Thus, the late-phase anti-EMT effect of PTEN does not appear to be linked to the function of PPARγ ligands.

### Exogenous treatment of cancer cells with ligands inhibits EMT via enhanced PPARγ/PTEN signaling

To confirm that 15-HETE, lipoxin A4, and 15d-PGJ_2_ act in a paracrine manner to induce anti-EMT effects through enhanced PPARγ/PTEN signaling, we investigated the effects of these soluble mediators on 344SQ cells at basal (80, 73, and 73 pg/ml, respectively) and stimulatory (258, 422, and 226 pg/ml, respectively) concentrations. The combination of these ligands at stimulatory concentrations enhanced PPARγ activity after 36 h, whereas this combination at basal concentrations exerted no effect (Supplementary Fig. S13a). As expected, each ligand partially inhibited the late-phase TGF-β1-induced EMT process at its stimulatory concentration but not at its basal concentration (Supplementary Fig. S13b and c). In parallel, the TGF-β1-induced reduction in the *PPARγ* mRNA abundance and activity (Supplementary Fig. S13d and e) and in the PTEN mRNA and protein abundances in 344SQ cells (Supplementary Fig. S13f and g) were reversed at stimulatory, but not basal, concentrations. In contrast, TGF-β1-induced non-Smad signaling, such as p38 MAP kinase and Akt phosphorylation, was not affected (Supplementary Fig. S13h and i).

### Apoptotic 344SQ cell treatment suppresses lung metastasis and enhances PPARγ/PTEN signaling

**Fig. 6:**
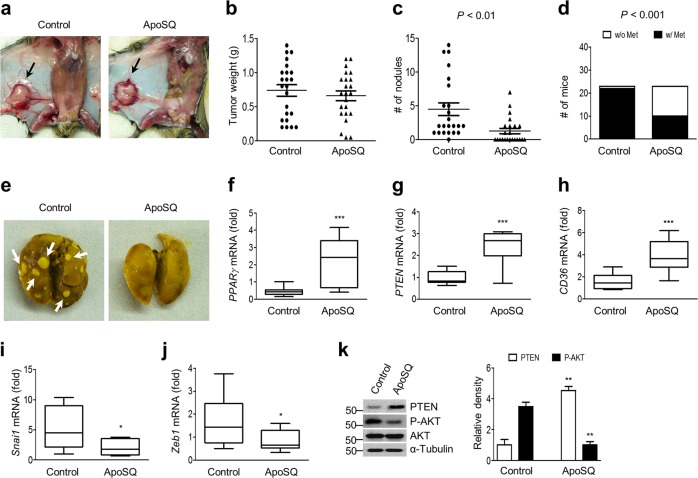
Antimetastatic effects of UV-irradiated apoptotic cancer cell injections in mice. Apoptotic 344SQ cells (ApoSQ) were subcutaneously injected into the skin lesion 2 days after subcutaneous injection of 344SQ cells into syngeneic (129/Sν) mice (*n* = 23 per group). Mice were necropsied 6 weeks later. **a** Representative images of primary tumors (black arrows). Scatter plots of primary tumor weight are shown in (**b**), and the numbers of metastatic pulmonary nodules are shown in (**c**). *P* values were determined by Student’s *t* test. **d** Bar graph indicates the number of mice with (w/) or without (w/o) visibly determined metastases (Met). The metastasis incidence *P* value (Fisher’s exact test). **e** Representative images of metastatic (*left*) or nonmetastatic lungs (*right*). White arrows indicate metastatic pulmonary nodules. The qPCR analysis is shown in (**f**–**j**), and immunoblot analysis of indicated protein expression is shown in (**k**) in primary tumors. *P < 0.05, **P < 0.01, and ***P < 0.001 (Student’s *t* test). The box represents the 25th to 75th percentile, and the whisker plots represent the minimum and maximum percentiles. Data are from mice with lung metastasis [control; *n* = 14 in (**f**–**j**) or *n* = 5 in (**k**)] and without lung metastasis [ApoSQ; *n* = 8 in (**f**–**j**) or *n* = 5 in (**k**)] (mean ± s.e.m. in (**f**–**k**))

To explore the effects of apoptotic lung cancer cells in mouse lung metastasis models, we injected syngeneic (129/Sν) immunocompetent mice subcutaneously with highly metastatic 344SQ cells and allowed them to grow for 6 weeks (Fig. 6a *left*). Apoptotic 344SQ (ApoSQ) administration 2 days after 344SQ cell injection did not significantly alter the primary tumor size (Fig. 6a *right* and b), but did diminish the tumor nodule number in the lungs and the number of mice with visible lung metastases 6 weeks after the 344SQ injection (Fig. 6c–e). ApoSQ injection increased the mRNA levels of *PPARγ* and its target molecules *PTEN* and *CD36*, as well as the PTEN protein level, but reduced the mRNA levels of *Snai1 and Zeb1* (Fig. 6f–j), as well as the Akt phosphorylation level (Fig. 6k).

**Fig. 7:**
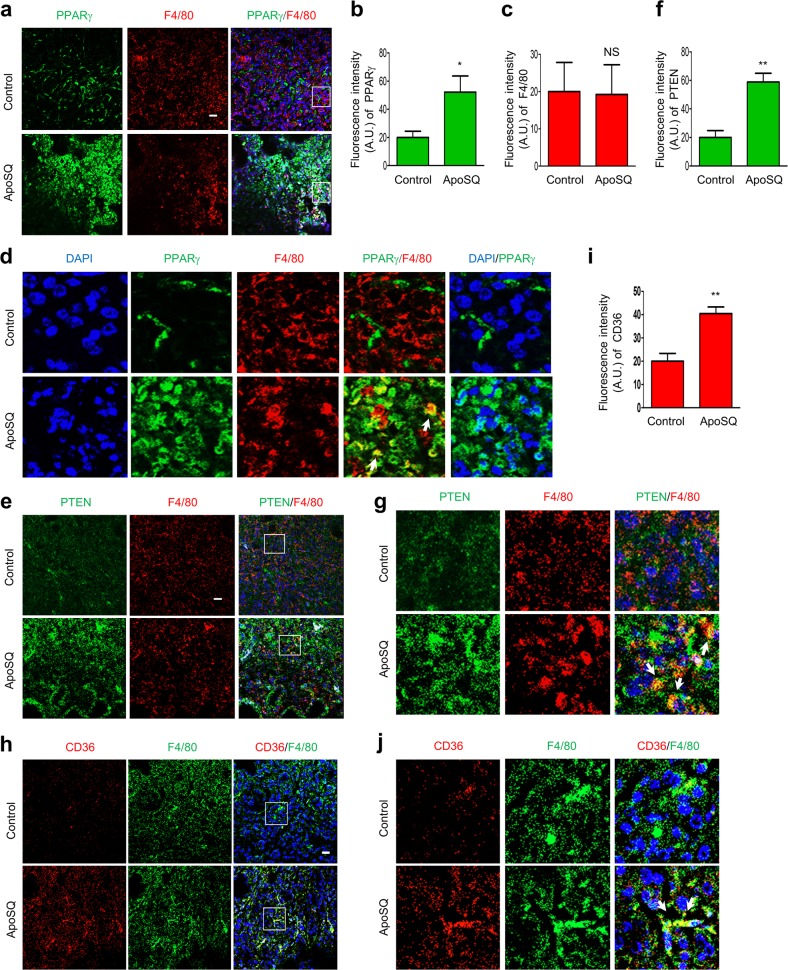
PPARγ and PTEN expression in primary tumors and tumor-infiltrating macrophages. Apoptotic 344SQ cells (ApoSQ) were subcutaneously injected into the skin lesion 2 days after subcutaneous injection of 344SQ cells into syngeneic (129/Sν) mice. Mice were necropsied 6 weeks later. Representative confocal images of primary tumors stained with anti-PPARγ (green) and anti-F4/80 (red) are shown in (**a**, **d)**; anti-PTEN (green) and anti-F4/80 (red) staining is shown in (**e**, **g**); and anti-CD36 (red) and anti-F4/80 (green) staining is shown in (**h**, **j**), as well as staining with the DNA-binding dye DAPI. **b**, **c**, **f**, **i** Measurements of fluorescence intensity in full-size images. **d**, **g**, **j** ROIs from white squares on the low magnification images of (**a**, **e**, **h**), respectively. Arrows indicate the localizations of PPARγ, PTEN, and CD36 in macrophages. NS not significant, ^*^P < 0.05 and ^**^P < 0.01. Data are representative images from five mice per group in (**a**, **e**, **h**) or from independent experiments with five mice per group (mean ± s.e.m. in (**b**, **c**, **f**, **i**)). Scale bars: 100 μm in (**a**, **e**, **h**)

Immunohistochemistry of serial sections of primary tumor tissue confirmed enhanced PPARγ (*green*, Fig. 7a, b), PTEN (*green*, Fig. 7e, f), and CD36 expression (*green*, Fig. 7h, i) upon ApoSQ injection. In particular, PPARγ (Fig. 7d), PTEN (Fig. 7g), and CD36 (Fig. 7j) expression in F4/80-positive cells (*red*) was also markedly enhanced by apoptotic cell injection, which reflects apparent PPARγ, PTEN, and CD36 induction in tumor-associated macrophages (TAMs), as the F4/80-positive macrophage intensity was not different (Fig. 7c).

**Fig. 8:**
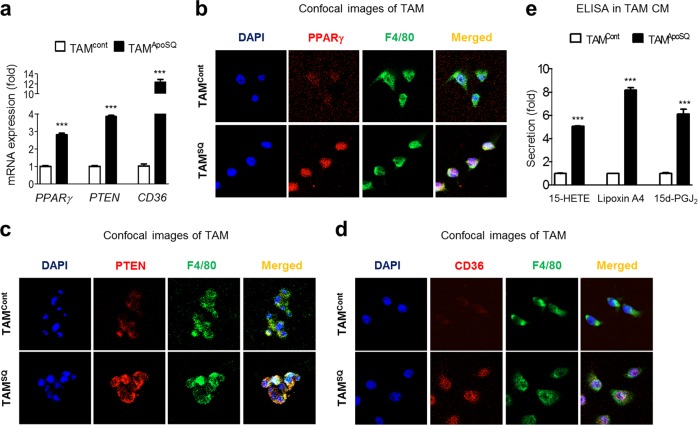
Expression of PPARγ and PTEN and the secretion of PPARγ ligands in isolated TAMs. **a** qPCR analysis of TAM isolated primary tumors (*n* = 8). **b**–**d** Representative confocal images of TAMs stained with anti-PPARγ (red), anti-PTEN (red), anti-CD36 (red), and anti-F4/80 (green). **e** ELISA of 15-HETE, lipoxin A4 and 15d-PGJ_2_ in TAM culture (*n* = 8). ***P < 0.001 (Student’s *t* test). Data are from mice with lung metastasis [control; *n* = 8 in (**a**–**e**)] and without lung metastasis [ApoSQ; *n* = 8 in (**a**–**e**)] (mean ± s.e.m. in (**a**, **e**))

Moreover, we found enhanced mRNA expression of *PPARγ* and its target genes, namely, *PTEN and CD36*, in TAMs isolated from primary tumors after ApoSQ injection compared to those isolated from control mice (Fig. 8a). Using confocal microscopy, we confirmed the enhanced protein expression of these molecules (red) in TAMs stained with F4/80 (green) (Fig. 8b–d). Interestingly, the secretion of PPARγ ligands, such as 15- HETE, lipoxin A4, and 15-PGJ_2_, was enhanced in the TAM culture media (Fig. 8e).

To investigate the early response of macrophages to ApoSQ injection in wild-type mice injected subcutaneously with 344SQ cells, with respect to PPARγ and PTEN induction, double immunofluorescence staining with PPARγ, PTEN, or F4/80 Ab was performed on cryosections derived from skin lesions. PPARγ (Supplementary Fig. S14a) and PTEN (Supplementary Fig. S14b) staining were markedly enhanced in F4/80-positive macrophages at 24 h following subcutaneous ApoSQ injection compared to the control group.

**Fig. 9:**
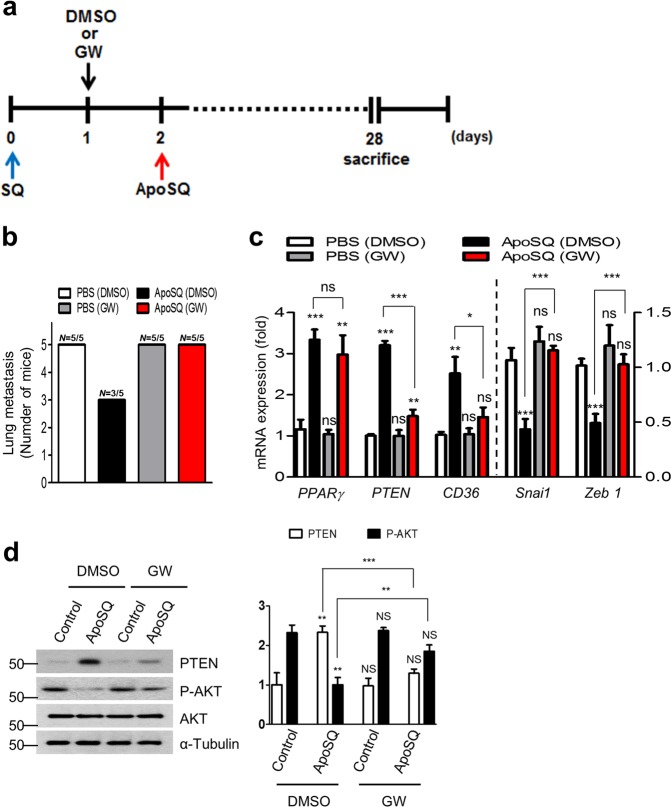
PPARγ-dependent anti-metastatic effects of UV-irradiated apoptotic lung cancer cell injection in mice. **a** A schematic of the experimental design. Where indicated, GW9662 (1 mg/kg/day, i.p.) or its vehicle (Veh; 2% DMSO in saline) was administered into the left flank 1 day before the injection of ApoSQ into the right skin lesion (*n* = 5 per group). Mice were necropsied 4 weeks after 344SQ cell injections. **b** Number of mice with lung metastasis/total number of mice examined. The qPCR analysis is shown in (**c**), and the immunoblot analysis of indicated protein expression in primary tumors is shown in (**d**). *P *<* 0.05, **P *<* 0.01, and ***P < 0.001 (Student’s *t* test). Data are representative images from five mice per group (**d** *left*) or from independent experiments with five mice per group (mean ± s.e.m. in (**c**, **d** *right*)

To confirm PPARγ-dependent PTEN expression and the concurrently mediated antimetastasis effect, the PPARγ antagonist GW9662 (1 mg/kg/d) was i.p. administered for 4 weeks, beginning 1 day before ApoSQ injection (Fig. 9a). GW9662 treatment reversed the reduction in the metastatic increase by ApoSQ injection (Fig. 9b). Interestingly, the enhancement of *PTEN* and *CD36* and the reduction in *Snai1* and *Zeb1* mRNA expression in the tumor tissue by ApoSQ injection were reversed by GW9662 treatment (Fig. 9c). Moreover, the ApoSQ-induced enhancement of PTEN protein expression and the reduction in Akt phosphorylation were also reversed by GW9662 treatment (Fig. 9d). This inhibitor administered with buffer had no effects.

## Discussion

EMT activation has previously been proposed as the critical mechanism in the acquisition of a malignant phenotype by epithelial cancer cells; we now propose that the interaction between macrophages and apoptotic lung cancer cells can provide an anticancer microenvironment that inhibits EMT and the multistep process of cancer cell dissemination.^42^ First, our in vitro data demonstrate that ApoSQ-exposed CM from RAW cells and primary mouse BMDMs inhibits TGF-β1-induced EMT in 344SQ cells. In addition, the interaction between macrophages and various types of apoptotic human cancer cells, such as non-small-cell lung, breast, colon, and prostate cancer cells, but not necrotic cells, results in the inhibition of EMT marker changes. These data clearly indicate that this anti-EMT effect is universal and specific. Similar to CM from wild-type mouse and human macrophages, CM from M2-like BMDMs that have TAM phenotypic properties and from blood MDMs of lung adenocarcinoma patients exposed to apoptotic lung cancer cells show anti-EMT effects.^43^These data suggest that macrophages under normal or cancerous circumstances may consistently have the ability to prevent EMT in response to apoptotic cancer cells.

Our data suggest that the downregulation of Smad-independent TGF-β1 signaling, as well as the p38 MAP kinase and Akt pathways, by ApoSQ-exposed CM inactivates transcription factors that bind to the *Snai1/2*, *Zeb1/2*, *and Twist1* promoters in the tumor microenvironment and consequently results in preventing EMT progression.^31^ Indeed, we found that apoptotic cancer cell-exposed CM inhibits the migration and invasion of lung cancer cells. Moreover, the anti-invasive effect of the ApoSQ-exposed CM was confirmed using 3D Matrigel culture. These findings provide new insight indicating that macrophages exposed to apoptotic cancer cells might generate a tumor microenvironment that prevents metastatic processes.

This is the first report of PPARγ activity-dependent PTEN induction in macrophages exposed to apoptotic, but not necrotic, lung cancer cells. Recently, two different groups demonstrated that PTEN secreted via exosomes, or PTEN-Long via an unknown mechanism, can be internalized by recipient cells.^35,44^ Surprisingly, we found enhanced canonical PTEN expression in PTEN immunoprecipitates from ApoSQ-exposed CM and confirmed the secretion of this protein via exosome formation. PTEN secretion in exosomes by human macrophages overexpressing GFP-PTEN in response to ApoA was also verified. Moreover, GFP fluorescence detection by using confocal microscopy and western blotting with anti-GFP antibodies demonstrated that PTEN-bearing exosomes were taken by recipient cancer cells. The way individual vesicles interact with recipient cells is still not known but has been proposed to involve binding to the cell surface via specific receptors, internalization through a variety of endocytic pathways or micropinocytosis, and/or fusion with the plasma membrane or with the limiting membranes of internal compartments.^45^ Accordingly, the PTEN level in recipient 344SQ cells was enhanced immediately and remained enhanced until 24 h after treatment with ApoSQ-exposed CM, with no changes in PTEN mRNA expression; Akt phosphorylation decreased reciprocally. Interestingly, ApoSQ-exposed CM from PTEN knockdown RAW cells failed to enhance the PTEN abundance or reduce TGFβ1-induced p38 MAP kinase or Akt phosphorylation. Taken together, these data indicate that enhanced PTEN protein levels in 344SQ cells do not originate from early-phase transcriptional induction but from their internalization into recipient cells with intact lipid and possible protein phosphatase activity.^46^

PTEN functions in a spatially restricted manner, which may explain its involvement in forming PIP3 gradients, which are necessary for generating and/or sustaining cell polarity in epithelial tissues.^38^ Accumulative evidence indicates that loss of cellular polarity and tissue architecture can drive tumor progression.^47^ Our data from the experiments using CM from PTEN knockdown RAW cells indicate that signaling through internalized PTEN mediates prolonged anti-EMT and anti-invasion effects in 344SQ cells, controlling early cell polarity and integrity by preventing the dissolution of cell–cell contacts. Importantly, we found that purified exosomes from ApoSQ-exposed CM have inhibitory effects on EMT and the Akt/p38 signal cascades in 2D cell culture and an anti-invasion effect in 3D Matrigel culture. These data confirm that internalized exosomal PTEN has functions in recipient cancer cells. The 3D culture of cancer cells provides an environmental condition that is closely related to in vivo conditions. Thus, our findings obtained from this 3D-culture model suggest that purified exosomes from ApoSQ-exposed CM would inhibit the early phase of metastasis. Future studies will show anti-metastasis effects of the isolated exosomes using a variety of in vivo models of metastasis. Moreover, treatment with purified exosomes from PTEN knockdown macrophages did not result in enhanced PTEN levels in recipient 344SQ cells within 24 h. Like treatment with ApoSQ-exposed CM, treatment with purified exosomes from ApoSQ-exposed CM did not alter the PTEN mRNA level in recipient 344SQ cells at 12 h. Taken together, these data suggest that the early enhancement of the PTEN protein level includes exosomal PTEN that is secreted from macrophagesm but that is not affected by PTEN mRNA transcription stimulated by certain molecules in exosomes. Nonetheless, further studies are needed to determine whether treatment with purified exosomes affects endogenous PTEN homeostasis, such as translation and degradation, in recipient cancer cells.

On the other hand, based on the prolonged effects of restored *PTEN* mRNA expression over 48 h following ApoSQ-exposed CM treatment, we propose that PTEN signaling in recipient 344SQ cells may originate from a different source, although the PTEN internalization rate, half-life, and stability of internalized PTEN were not estimated.^48^ Notably, with regard to PPARγ activation over 72 h after treatment with ApoSQ-exposed CM, 344SQ cells demonstrated a striking resemblance to cells with *PTEN* mRNA expression, with dependence on PPARγ activity. These data support the novel insight that enhanced late-phase *PTEN* mRNA expression may be induced mainly through PPARγ-dependent transcriptional upregulation in recipient lung cancer cells.

We observed enhanced secretion of PPARγ ligands, such as 15-HETE, lipoxin A4, and 15d-PGJ_2_, in apoptotic 344SQ-exposed CM but not in viable or necrotic 344SQ-exposed CM. Interestingly, ApoSQ-exposed CM deficient in these PPARγ ligands partially failed to reverse the reductions in *PPARγ* mRNA and activity and the PTEN mRNA and protein levels in recipient 344SQ cells, and consequently, EMT was not effectively prevented. These data suggest that ligand-dependent PPARγ/PTEN signaling in 344SQ cells also mediates the anti-EMT effects of ApoSQ-exposed CM. Supporting this hypothesis, the exogenous addition of these lipid mediators to 344SQ cells partially reversed the TGFβ1-induced reductions in *PPARγ* mRNA expression and activation and in PTEN mRNA and protein expression and that these mediators concomitantly inhibited EMT. Unlike ApoSQ-exposed CM, these mediators did not affect early-phase TGFβ1-induced Akt or p38 MAP kinase phosphorylation, indicating that their anti-EMT effects bear no relation to early signaling events.

Increasing evidence indicates that PTEN loss triggers EMT in many cancer cell types and consequently promotes the invasion and metastasis of various cancers.^6,8,9^ In several xenograft models, intraperitoneal injection of PTEN-Long (a translational variant of PTEN) leads to tumor regression, which is dependent on PTEN-Long phosphatase activity.^44^ Notably, PTEN deletion in stromal fibroblasts accelerates the initiation, progression, and malignant transformation of mammary epithelial tumors.^11^ Here, we demonstrated that a single administration of ApoSQ around the lesion 2 days after 344SQ cell injection into syngeneic mice diminished number of metastatic nodules and the lung metastasis incidence in vivo. However, some mice treated with ApoSQ developed lung metastases after 6 weeks of treatment with 344SQ cells. ApoSQ use should be modulated; more injections, modifications to injection timing, combined therapy with agents enhancing apoptotic cell clearance, or PTEN-bearing exosome therapy with PPARγ ligands might be needed. Importantly, a single-ApoSQ injection leads to the enhanced induction of *PPARγ* and PTEN mRNA and protein expression and to a reciprocal reduction in phosphorylated Akt, as well as in the mRNA levels of *Snai1 and Zeb1*, within primary tumor tissue. Importantly, confocal microscopic analysis confirms apparent PPARγ and PTEN induction in tumor cells and infiltrating macrophages. Not surprisingly, increases in PPARγ, PTEN, and CD36 mRNA or protein expression were also shown in TAMs isolated from primary tumors after ApoSQ injection. Additionally, enhanced production of PPARγ ligands, such as 15-HETE, lipoxin A4 and 15d-PGJ_2_, was detected in the culture media of TAMs. These in vivo and ex vivo data suggest that the early injection of apoptotic cancer cells results in a shift in TAMs to an antimetastatic phenotype, leading to the PPARγ-dependent production of PTEN and its ligands. However, direct evidence is not provided to support the notion that TAMs are the targets of apoptotic cells in suppressing tumor metastasis in vivo. Thus, the possible roles of exosomal PTEN and PPARγ ligands secreted from TAMs upon apoptotic cancer cell stimulation in inhibiting EMT in an in vivo model need to be investigated. Using PPARγ antagonist GW9662, we confirmed PPARγ-dependent PTEN signaling, as well as a reduction in *Snai1* and *Zeb1* mRNA expression and their concurrent mediation of the anti-metastasis effect.

In addition to macrophages, apoptotic cells can also target other cells, such as dendritic cells (DCs), to cross-prime CD8 T cells.^49^ A recent study has shown that dying hepatocellular, colorectal, and breast cancer cells, induced by chemotherapy and radiotherapy, can enhance the maturation and antigen presentation of DCs.^50^ In addition, DCs charged with apoptotic tumor cells induce long-lived protective CD4^+^ and CD8^+^ T cell immunity against B16 melanoma in vivo.^51^ Nonetheless, a role for tumor-infiltrating DCs in preventing cancer progression and metastasis following apoptotic cancer cell administration remains to be identified.

Notably, TAMs may have a dual role in terms of interfering with cancer treatments, as TAMs can either promote or impair the functionality these treatments.^52^ Many studies have demonstrated a role for TAMs in supporting multiple aspects of tumor progression.^53^ Interestingly, tumor-conditioned macrophages have been shown to produce migration-stimulating factor, which strongly stimulates tumor cell migration, thus contributing to the motile phenotype of tumor cells.^54^ However, our data demonstrate that when TAMs are in contact with apoptotic cancer cells, they can play an antitumor role, through inhibition of EMT, migration and invasion, in cancer cells.

Within a tumor environment where rapid cell proliferation and apoptosis are ongoing, apoptotic cell clearance can exert anti-inflammatory and tolerogenic properties that suppress innate and adaptive antitumor immune responses.^55^ On the other hand, the therapeutic use of apoptotic cells needs to be carefully considered in cases in which the capacity for apoptotic cell clearance is reduced in vivo, as administered cells may progress into secondary necrosis, which can exacerbate inflammation or autoimmunity.^56^ To prevent these potential unwanted responses against apoptotic cells, alternative noncell-based therapies, such as CM from macrophages exposed to apoptotic cancer cells, can be used. Thus, future studies are necessary to evaluate whether in vivo treatment with the CM has therapeutic feasibility against cancer progression and metastasis.

**Fig. 10:**
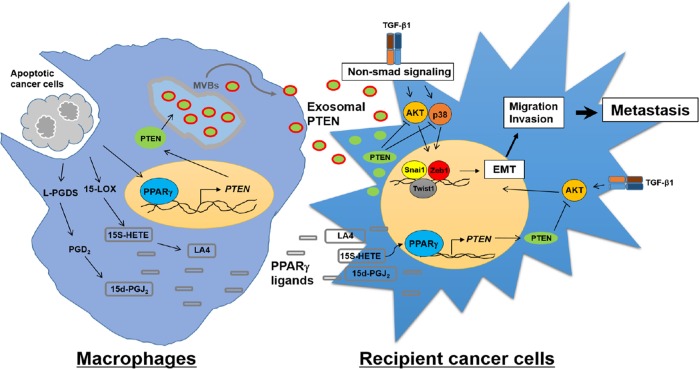
A schematic diagram summarizing and integrating the effects of enhanced exosomal PTEN secretion and PPARγ ligands from macrophages exposed to UV-irradiated apoptotic cancer cells to prevent cancer cells from undergoing the EMT and metastatic process

In summary, we propose that PTEN secretion in exosomes from macrophages exposed to UV-irradiated apoptotic lung cancer cells can be internalized into recipient cancer cells, inhibiting cell polarity disruption, EMT, and invasion. In addition, the secretion of PPARγ ligands from macrophages may reinforce tissue homeostasis and the defense against cancer progression via enhanced PPARγ/PTEN signaling in cancer cells. Thus, our results indicate that programming macrophages with UV-irradiated apoptotic lung cancer cells might create a tumor microenvironment that antagonizes cancer progression and lung metastasis via prolonged PPARγ/PTEN signaling in both TAM and tumor cells. A mechanistic diagram summarizing and integrating the effects of enhanced exosomal PTEN secretion and PPARγ ligands from macrophages exposed to UV-irradiated apoptotic cancer cells to prevent the cancer cells from undergoing EMT and the metastatic process is shown in Fig. 10. Importantly, our studies provide new opportunities to develop apoptotic cancer cell therapy or alternative non-cell-based therapies, such as the CM/CM compositions that include exosomal PTEN and PPARγ ligands, as an effective antimetastasis tool in a variety of experimental and clinical settings.

## Materials and methods

### Reagents

GW9662 (#70785) and PD146176 (#10010518) were purchased from Cayman Chemical. GW4869 (#6823-69-4) was purchased from Sigma-Aldrich. TGF-β1 (240-B-010) and IL-4 (404-ML) were purchased from R&D Systems. 15-HETE and 15d-PGJ_2_ were purchased from Enzo Life Sciences. Lipoxin A4 was purchased from Neogene. Mouse IgG Blocking Reagent (MKB-2213) was purchased from Vector Laboratories.

### Antibodies

The antibodies used for western blotting and immunofluorescence are listed in Table S1.

### Cell lines, primary cells, and culture

Murine RAW 264.7 cells and human cancer cell lines were obtained from ATCC (American Type Culture Collection). 344SQ cells (gift from Dr. Kurie) and the various human cancer cell lines [A549 (lung), MDA-MB-231 (breast), COLO320HSR (colon), and PC3 (prostate)] were maintained in RPMI 1640 (HyClone^TM^, GE Healthcare) containing 10% fetal bovine serum (FBS) and 1% penicillin/streptomycin.^47^ RAW 264.7 cells were grown in DMEM (Gibco^TM^, Thermo Fisher Scientific) supplemented with 10% FBS and 1% penicillin/streptomycin. Bone marrow cells from C57BL/6 mice were cultured in DMEM supplemented with 10% FBS and 20% L929 supernatant (BMDM medium) for 7 days. For differentiation of M2-like cells, BMDMs were grown in 50 ng/ml IL-4-containing BMDM media for 3 days.^57^

### Blood samples from patients

Lung cancer patients and healthy controls were included in the study after informed consent under protocols approved by the Institutional Review Board of Ewha Womans University, School of Medicine. A total of three healthy control subjects (one male and two females) and three non-small-cell lung cancer patients without anticancer drugs (one male and two females) were used in the experiments depicted in Figs. 1d and 2c and Supplementary Fig. S3g, h. Human monocytes were collected from 20 ml of blood by Ficoll-Histopaque density gradient centrifugation.^58^ Purified monocytes were grown in RPMI containing 10% human AB serum for 8 days. The confirmation of monocyte differentiation into macrophages (MDM) was performed by confocal microscopy with anti-F4/80.

### Conditioned medium

Murine macrophages (RAW, BMDM, and M2-like cells) or human blood MDM were plated at 5 × 10^5^ cells/ml and grown in suitable medium (refer to Cell culture) at 37 °C and 5% CO_2_. After overnight incubation, the cells were serum-starved with X-VIVO 10 medium (04-380Q, Lonza) for 24 h before cell stimulation. For the stimulation, the culture medium was replaced with X-VIVO 10 containing apoptotic or necrotic cancer cells (1.5 × 10^6^ cells/ml). After 24 h, supernatants were harvested by centrifugation and used as the CM for the stimulation of target cancer epithelial cells (5 × 10^5^ cells/ml).

### Induction of cell death

Cancer epithelial cell lines were exposed to ultraviolet irradiation at 254 nm for 10 min followed by incubation in RPMI-1640 with 10% FBS for 2 h at 37 °C and 5% CO_2_. Evaluation of nuclear morphology using light microscopy on Wright-Giemsa-stained samples indicated that the irradiated cells were nearly apoptotic.^59^ Lysed (necrotic) cancer cells were obtained by multiple freeze-thaw cycles.^60^ Apoptosis and necrosis were confirmed by annexin V-FITC/propidium iodide (BD Biosciences, San Jose, CA, USA) staining followed by flow cytometric analysis on a FACSCalibur system (BD Biosciences).^59^ Supplementary Fig. S15a–d shows representative dot plots depicting the percentages of apoptotic and necrotic 344SQ and A549 cells.

### Immunoprecipitation

Sixty milliliters of CM was prepared with 1 × 10^6^ per ml of BMDMs that had been stimulated with nonapoptotic or apoptotic 344SQ cells. In the case of unconventional secretion of PTEN, the medium was diluted 1:1 with 2× exosome lysis buffer (4% SDS, 2% Triton-X100, 0.1 M Tris pH 7.4 and 2× protease inhibitors) and lysed for 1 h at 4 °C.^61,62^ The resulting medium-lysis buffer mixture was filtered through a 0.22-micron filter (Macherey-Nagel) and divided into 15-ml vials. Twenty-five microliters of anti-PTEN (138G6, Cell Signaling) was added to a mixture vial and incubated for 4 h with rotation at 4 °C. Immunocomplexes were then precipitated with 100 μl (50% slurry) of Protein A/G Sepharose (BioVision Inc.). For the remaining mixture vials, the pull-down beads with the prebound immunocomplexes were added to new tubes and incubated for 4 h with rotation at 4 °C repeatedly. They were then washed four times with IP wash buffer (25 mM HEPES pH 7.4, 1 M NaCl, 1 mM EDTA, 0.5% Triton X-100). To avoid overlap with IgG heavy chains, western blotting for PTEN immunoprecipitates was performed in nonreducing conditions.

### Western blotting

Standard western blotting was performed using whole-cell extracts, (in)soluble fractionates from conditioned media, or immunoprecipitates. The information for the antibodies used in included in Table S1.

### Real-time quantitative polymerase chain reaction (qPCR)

mRNA was extracted from cells grown to 80% confluence in triplicate on six-well plates under experimental conditions and quantified using a Real-Time PCR System (Applied Biosystems, Step One Plus). The primer sequences of the target genes are provided in Table S2.

### Migration and invasion assays

Cell migration and invasion were examined using Transwell chambers (Corning Inc.) coated with 10 μg/ml fibronectin and 200 μg/ml Matrigel matrix according to the respective manufacturer’s instructions. In brief, preincubated cancer cells (5 × 10^4^ cells/well for the migration assay and 2 × 10^5^ cells/well for the invasion assay) in CM from macrophages plus or minus TGF-β1 (10 ng/ml) were seeded to the upper chambers of replicate wells in serum-free RPMI, and RPMI 1640 supplemented with 10% FBS was added to the bottom wells; cells were incubated at 37 °C for 16 h for migration assays or 24 h for invasion assays. After fixation in 4% PFA, the nonmigrated or noninvaded cells on the upper surface of the membrane were scraped off with a cotton swap. The cells on the lower surface were stained using 0.1% crystal violet and washed with distilled water. Three random microscopic fields (×10 magnification) were photographed and counted.

### 3D cell culture on Matrigel

Standard 3D culture was performed as described previously.^63^ Briefly, a single-cell suspension containing 5000 cells/well was added to the top layer of the solidified Growth Factor Reduced Matrigel (Corning Inc.) in an 8-well plate. The cells in RPMI 1640 with 10% FBS and 2% Matrigel were incubated, and the medium was changed every 2 or 3 days for 1 week. After incubation, the cells were treated with the indicated CM containing TGF-β1 (10 ng/ml) and 2% Matrigel and then grown for 3 days. Phase-contrast images were taken using an Eclipse TE-300 microscope (Nikon).

### Immunofluorescence

344SQ cells were grown on glass coverslips until confluent and fixed with 4% paraformaldehyde (PFA) solution for 8 min at room temperature (RT). For the staining of 344SQ acini in the Matrigel 3D culture, paraffin-embedded tumor tissues or frozen skin tissues, formalin fixation was performed at RT for 30 min and IF-Wash buffer (0.05% NaN3, 0.1% BSA, 0.2% Triton X-100 and 0.05% Tween-20 in PBS) was used. After fixation, samples were washed three times with wash buffers for 5 min each and permeabilized with 0.5% Triton X-100 in PBS at RT for 5 min. Five percent bovine serum albumin in PBS and PBS containing Mouse IgG Blocking Reagent were used for ICC and IHC, respectively. Subsequently, all slides stained with antibodies were mounted with Vectashield Mounting Medium containing DAPI (Vector Laboratories, Inc.) and imaged with a confocal microscope (LSM 800, Carl Zeiss). Information on the antibody sources or dilution ratios is provided in Table S1.

### siRNA transfection

RAW 264.7 cells were transiently transfected with specifically targeted siRNA or control siRNA (SN-1003_AccuTarget^TM^ Negative Control; Bioneer Inc) at a final concentration of 100 nM using the GeneSilencer^Ⓡ^siRNA Transfection Reagent (Genlantis Inc) according to the manufacturer’s instructions. After overnight transfection, the cells were cultured in suitable medium for 24 h and stimulated with apoptotic 344SQ cells. The siRNA sequences used for gene targeting were as follows: (gene: sense, antisense). PPARγ (#1): 5′-AGUAUGGUGUCCAUGAGAU-3′, 5′-AUCUCAUGGACACCAUACU-3′; PPARγ (#2): 5′-CUGGUUUCAUUAACCUUGA-3′, 5′-UCAAGGUUAAUGAAACCAG-3′; PTEN (#1): 5′-CAGGAAUGAACCAUCUACA-3′, 5′-UGUAGAUGGUUCAUUCCUG-3′; PTEN (#2): 5′-CUGAGUAGAAACAAGAGUA-3′, 5′-UACUCUUGUUUCUACUCAG-3′; L-PGDS (#1): 5′-CAACUAUGACGAGUACGCUCUGCUA-3′, 5′-GACUUCCGCAUGGCCACCCUCUACA-3′; and L-PGDS (#2): 5′-GAAGAAAGCUGUAUUGUAU-3′, 5′-AUACAAUACAGCUUUCUUC-3′.

### Detection of PPARγ ligands

The concentrations of PPARγ ligands in the CM were measured using ELISA kits [15-HETE (ADI-900–051, Enzo Life Sciences), lipoxin A4 (#407010, Neogene), PGD_2_ (MBS703802, MyBioSource) and 15d-PGJ_2_ (ADI-900–023, Enzo Life Sciences)] according to the manufacturer’s protocols.

### PPARγ activity assay

PPARγ activity was determined in nuclear extracts (8 g) frompharmacological inhibitor-pretreated or siRNA-transfected RAW264.7 or 344SQ cells using a TransAM^TM^ PPARγ Transcription Factor Assay kit (40196, Activ Motif Inc.) according to the manufacturer’s instructions.

### Exosome purification

Exosomes were isolated from cell culture media by differential centrifugation as described previously.^64^ In brief, supernatant from RAW264.7 cells exposed to apoptotic or necrotic 344SQ cells was subjected to serial centrifugation at 200 *g*, 20,000 *g*, and 100,000 *g* for the clearance of dead cells, cell debris, and nonexosomal fraction. After washing the exosome pellet with ice-cold PBS containing protease inhibitors, the ultracentrifugation (Optima L-100K, Beckman Coulter Inc., Brea, CA, USA) was repeated for 70 min at 100,000 *g* to eliminate contaminating proteins. The exosome pellet was then resuspended in RIPA buffer containing protease inhibitors. Western blotting analysis was performed for the identification of exosomal PTEN in CM with anti-CD63 (ab216130, Abcam) or anti-PTEN (9559, Cell Signaling Technology). For cell treatments, 30 ml of CM from macrophages with or without apoptotic cancer cells in two 150-mm culture dishes was clarified by differential ultracentrifugation. Insoluble pellets were resuspended in 100 μl of sterilized 1× PBS, and 20 μl of this pellet was treated with recipient cancer cells for each experimental condition.

### Transmission electron microscopy

Exosomes in 10-µl aliquots were fixed in 4% PFA for 1 h at RT. Six-microliter aliquots of fixed exosome solution were applied to copper mesh Formvar-coated carbon stabilized grids, allowed to adsorb to the grid for 5 min and wicked off with filter paper, followed by analysis using TEM (H-7650; Hitachi) at an accelerating voltage of 80 kV. iTEM software (Olympus) was used for image acquisition.

### Nanoparticle tracking analysis

NTA was performed using the NanoSight NS300 system (Malvern Instruments, UK).^65^ The NanoSight polystyrene latex calibration beads, 100 and 200 nm, were applied to check the instrument performance. The size of the exosomes was determined based on both light scattering and Brownian motion and calculated using the Stokes–Einstein equation with NTA 3.0 analytical software (Malvern). NTA software was used to measure the concentration of the particles (particles/ml) and size distribution (in nm). Each sample was measured three times.

### Generation of stable macrophages overexpressing GFP-PTEN

Standard lentiviral transduction was performed as described previously.^66^ In brief, HEK293T cells were cotransfected with pLV-EGFP-PTEN, a packaging vector (psPAX2) and an envelope (pCMV-VSV-G) vector using the TransIT^Ⓡ^-LT1 Transfection Reagent (MIR 2300, Mirus Bio, Madison, WI, USA) according to the manufacturer’s instructions and incubated overnight. For lentiviral transductions, the reagents were replaced with fresh media, and the viral supernatants were collected every 24 h after transfection. Supernatants were exposed to human macrophages (hMϕ) differentiated from THP-1 monocytes plus 8 µg/ml polybrene for 4 h every time a virus collection was performed, and the infected cells were selected with 1 μg/ml of puromycin-containing media.^67^

### Detection of exosomal GFP-PTEN in recipient cells

GFP-PTEN-overexpressing THP-1 macrophages were seeded in ten 150-mm tissue culture dishes and grown to 70% confluency. After stimulation with apoptotic A549 cells for 24 h, conditioned media were collected and subjected to serial centrifugation (refer to exosome purification). The exosome pellet was resuspended in 200 μl of serum-free RPMI. Recipient cells (A549 or 344SQ, 1 × 10^5^ cells for confocal microscopy or 3 × 10^5^ cells for western blotting analysis) were exposed to 50 μl of purified exosomes for 24 h. GFP-PTEN in recipient cells was visualized by direct fluorescence and detected with anti-GFP using whole-cell lysates.

### Mouse experiments

The Animal Care Committee of the Ewha Medical Research Institute approved the experimental protocol. Mice were cared for and handled in accordance with the National Institute of Health (NIH) Guide for the Care and Use of Laboratory Animals. The syngeneic tumor experiments for the lung cancer metastasis studies were performed as previously described.^68^ In brief, syngeneic (129 Sv) mice (*n* = 23 per group) that were at least 8 weeks old were used for the syngeneic tumor experiments. A total of 1 × 10^6^ 344SQ cells in a single-cell suspension were subcutaneously injected into the right posterior flank. Two days after the first injection, a second injection of 100 μl of PBS with or without 1 × 10^7^ apoptotic 344SQ cells was performed in the same lesion. Mice were monitored daily for tumor growth and sacrificed at 6 weeks after injection. Necropsies were performed to investigate the weights of the subcutaneous tumor masses and the lung metastatic status (# of nodules or incidence), and histological evaluations of formalin-fixed, paraffin-embedded, immunofluorescence-stained primary tumors were performed. For the inhibition experiments, the selective PPARγ antagonist GW9662 (1 mg/kg/d) was i.p. administered for 4 weeks, beginning one day before the ApoSQ injection. Necropsies of the mice were performed 4 weeks after the 344SQ cell injection.

### TAM isolation

Purification of TAM was performed as described previously.^69^ To generate single-cell suspensions from the lung tumors of metastatic mouse models with or without the injection of ApoSQ (*n* = 8 per group, refer to mouse experiments), solid fresh cancer tissues were disaggregated with tumor digestion medium containing Collagenase I and IV and DNase I. After filtration with 70 μm of sterile nylon gauze, red blood cells were lysed with erythrocyte lysis buffer. Density gradient centrifugation was performed to isolate mononuclear cells from sharp interphase, and sedimented cells (mostly cancer cells) were used for western blotting analysis. From mononuclear cells washed with MACS buffer, TAMs were isolated using anti-CD11b^+^-conjugated magnetic beads and MACS columns (Miltenyi Biotec).

### Statistics

Comparisons between two mean values ± SEM (control vs. experimental) were performed using a two-tailed Student’s *t* test. *P* values that were less than 0.05 were considered statistically significant. All data were analyzed using GraphPad Prism 5 software (GraphPad Software Inc.).

## Supplementary information

Supplementary Materials
