# Supplementary material for: Programming of macrophages by UV-irradiated apoptotic cancer cells inhibits cancer progression and lung metastasis
Source: Cell Mol Immunol. 2019 Mar 6;16(11):851–67. doi: 10.1038/s41423-019-0209-1 (PMC6828747; doi:10.1038/s41423-019-0209-1)

**Supplementary information**

**Programming of macrophages by UV-irradiated apoptotic cancer cells inhibits cancer progression and lung metastasis**

Yong-Bae Kim, Young-Ho Ahn, Ji-Hae Jung, Ye-Ji Lee, Jin-Hwa Lee, and Jihee Lee Kang*

^*^

*Correspondence should be addressed to:

[jihee@ewha.ac.kr](mailto:jihee@ewha.ac.kr)

**Supplementary figures**

**
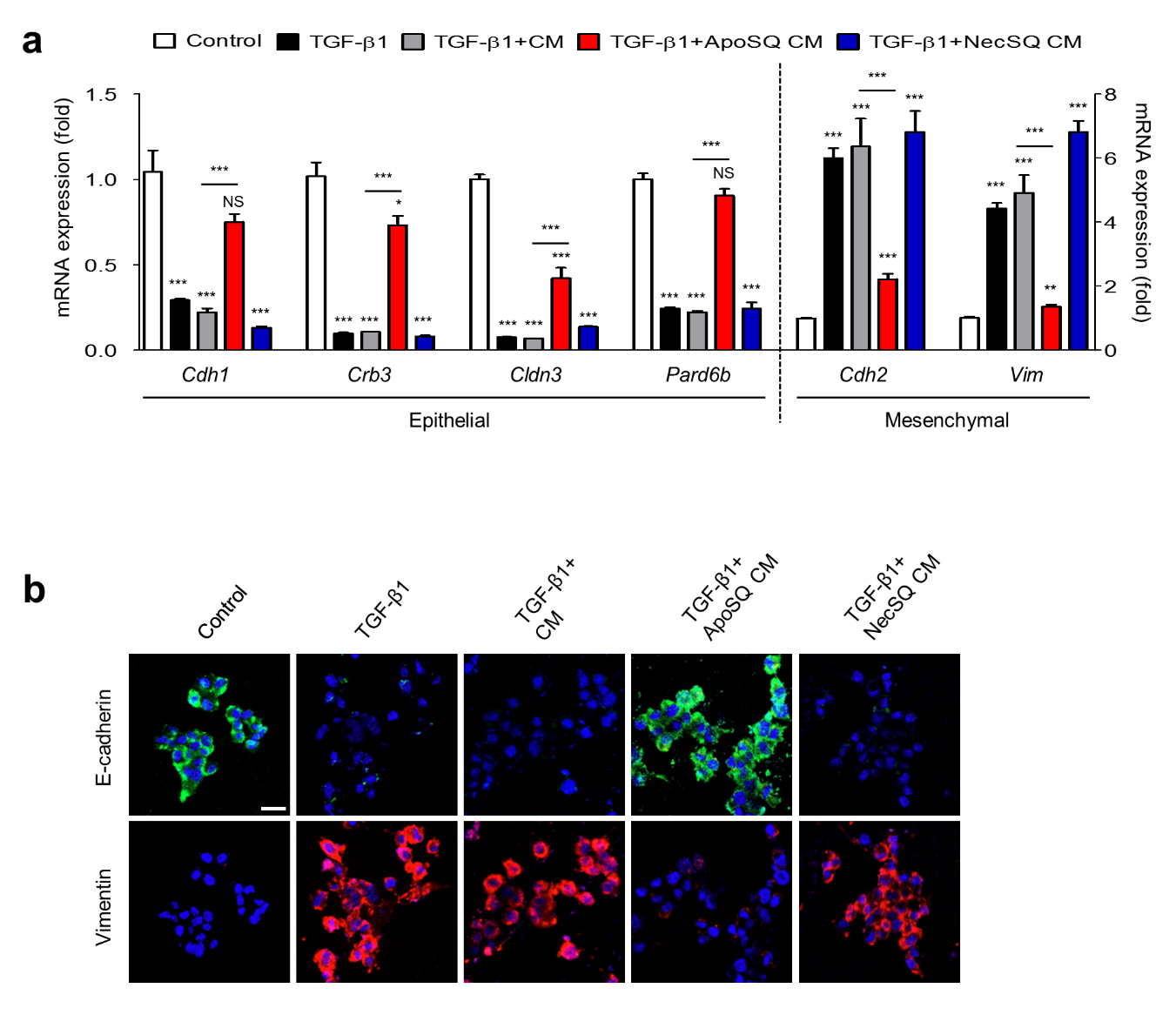
**

**Supplementary Fig. S1** Interaction of macrophages and apoptotic lung cancer cells inhibits EMT in cancer cells. **a** Real-time PCR analysis of the indicated epithelial markers and mesenchymal markers in 344SQ samples. **b** Immunofluorescence staining for E-cadherin (greed) or vimentin (red) in 344 SQ cellss. Scale bars = 20 μm. **a** and **b** RAW cells were incubated with apoptotic or necrotic 344SQ cells (ApoSQ or NecSQ). After 24 h, conditioned medium (CM) was added to the corresponding cancer cells with or without TGF-β1 (10 ng/ml) for 48 h. NS, not significant; **P* < 0.05, ***P* < 0.01 and ****P* < 0.001. Data are from three independent experiments (mean ± s.e.m. in **a**), or from one experiment representative of three independent experiments with similar results in **b**.

**
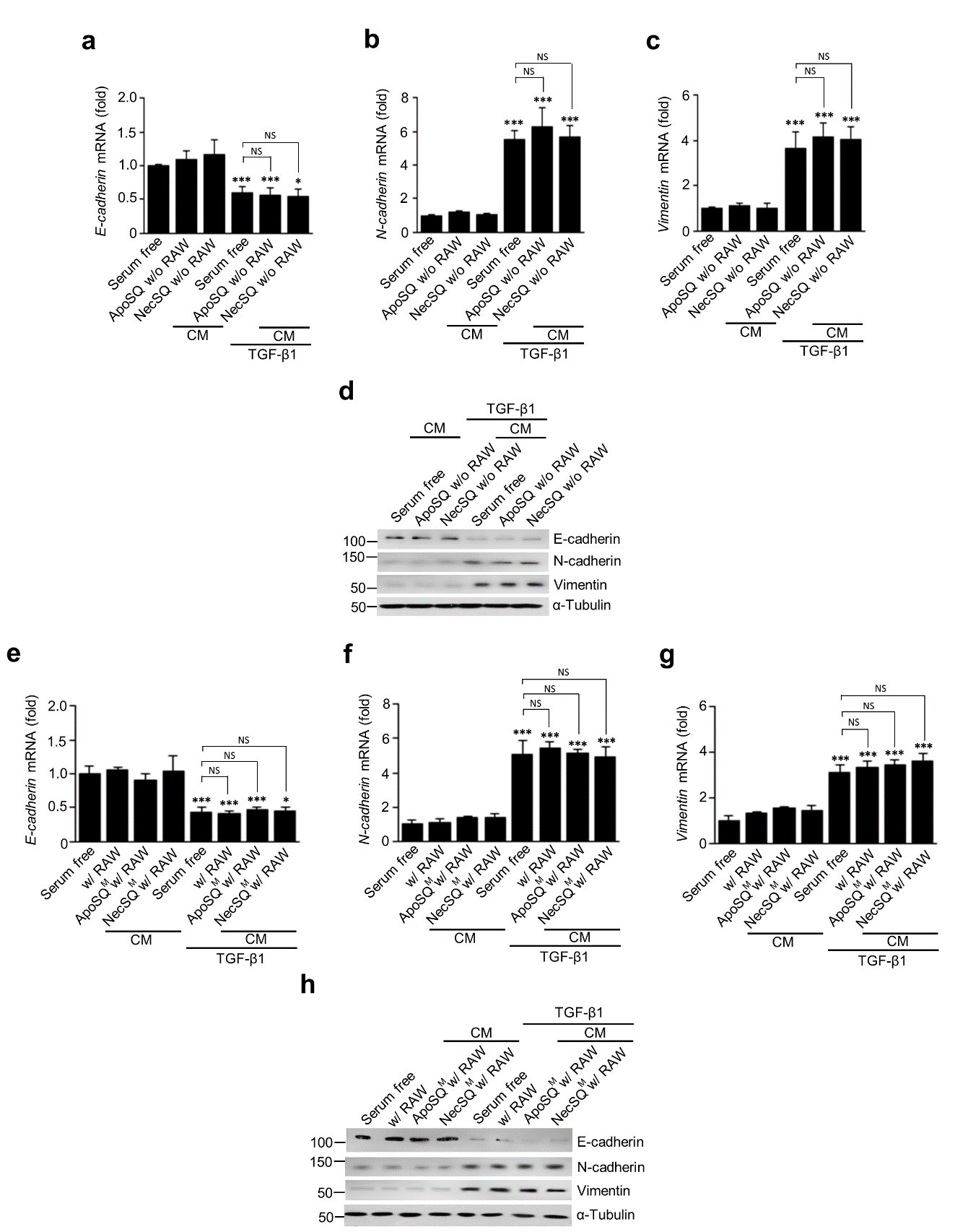
**

**Supplementary Fig. S2** Effect of control media from apoptotic cancer cells on TGF-β1-induced EMT. **a-c** and **e-g** Real-time PCR analysis of the indicated EMT markers in 344SQ samples. **d** and **h** Immunoblot analysis of indicated EMT markers in 344SQ cell lysates. **a-d** ApoSQ or NecSQ were incubated without RAW cells. After 24 h, the medium was added to the 344SQ cells with or without TGF-β1 (10 ng/ml) for 48 h. **e-h** RAW cells incubated with the medium of ApoSQ or NecSQ alone for 24 h. After 24 h, CM was added to the 344SQ cells with or without TGF-β1 (10 ng/ml) for 48 h. NS, not significant; **P* < 0.05, ***P* < 0.01 and ****P* < 0.001. Data are from three independent experiments (mean ± s.e.m. in **a-c,** and **e-g**), or from one experiment representative of three independent experiments with similar results in **f** and **h**.


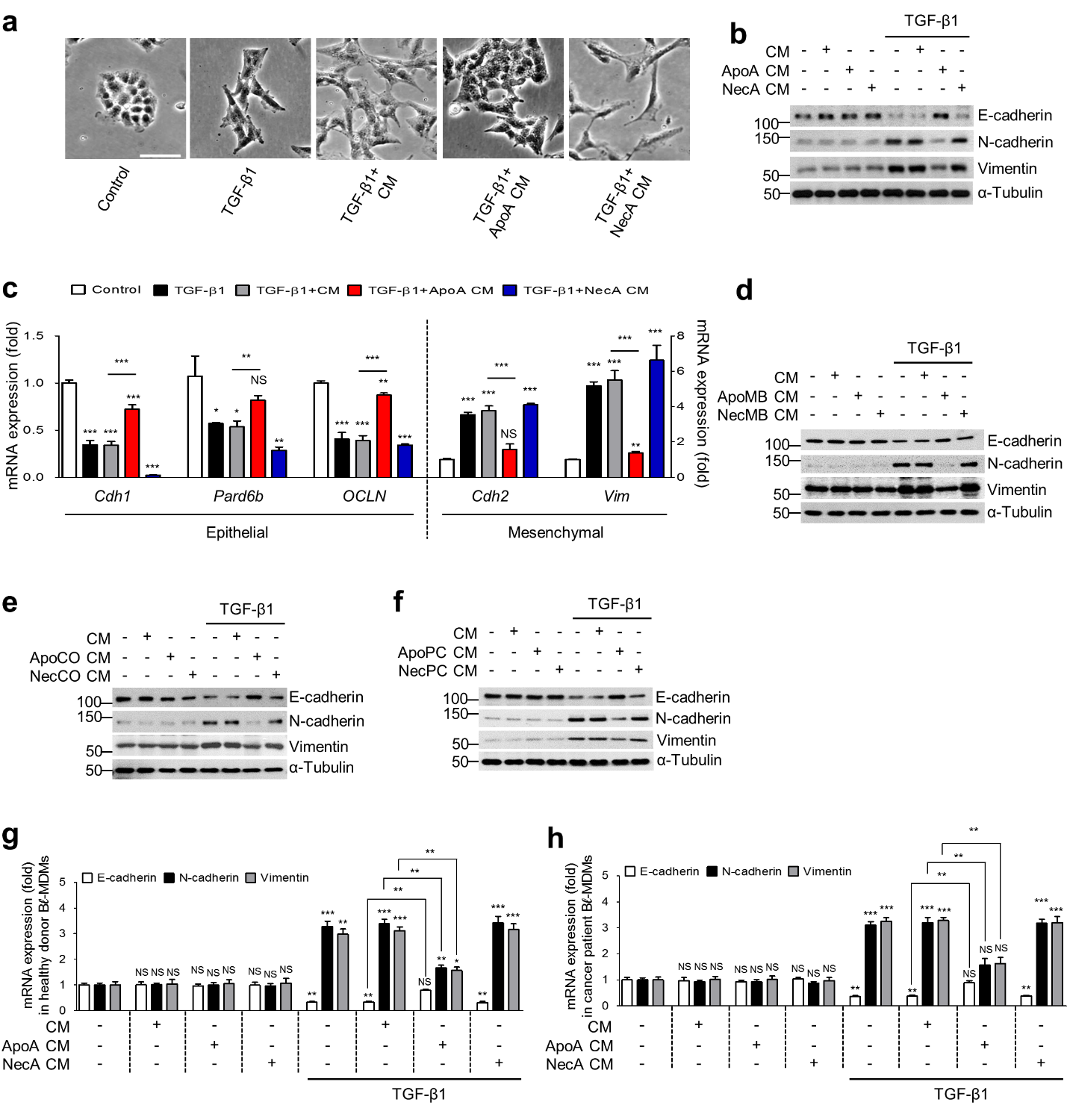


**Supplementary Fig. S3** Interaction of macrophages and apoptotic cancer cells inhibits EMT in cancer cells. **a** Morphological changes in A549 cells were examined by phase-contrast microscopy. Scale bar, 100 μm. **b** Immunoblot analysis of indicated EMT markers in A549 cell lysates. **c** Real-time PCR analysis of the indicated epithelial markers and mesenchymal markers in A549 samples. **d** Immunoblot analysis of indicated EMT markers in MDA-MB-231 cell lysates. **e** Immunoblot analysis of indicated EMT markers in COLO320HSR cell lysates. **f** Immunoblot analysis of indicated EMT markers in PC3 cell lysates. **g and h** Real-time PCR analysis of the indicated EMT markers in A549 samples. RAW cells were stimulated with apoptotic cancer cells, such as A549 (ApoA) in **a-c**, MDA-MB-231 (ApoMB) in **d**, COLO320HSR (ApoCO) in **e**, and PC3 (ApoPC) in **f**, or necrotic cancer cells (Nec A, NecMB, NecCO, and NecPC, in **a-c, d, e,** and **f**, respectively). Blood monocyte-derived macrophages (MDMs) from healthy donors in **g** or lung cancer patients in **h** were stimulated with ApoA or NecA. **a-h** After 24 h, conditioned medium (CM) was added to the corresponding cancer cells with or without TGF-β1 (10 ng/ml) for 48 h. NS, not significant; **P* < 0.05, ***P* < 0.01 and ****P* < 0.001. Data are from one experiment representative of three independent experiments with similar results in **a, b** and **d-f**, or from three independent experiments (mean ± s.e.m. in **c, g** and **h**).


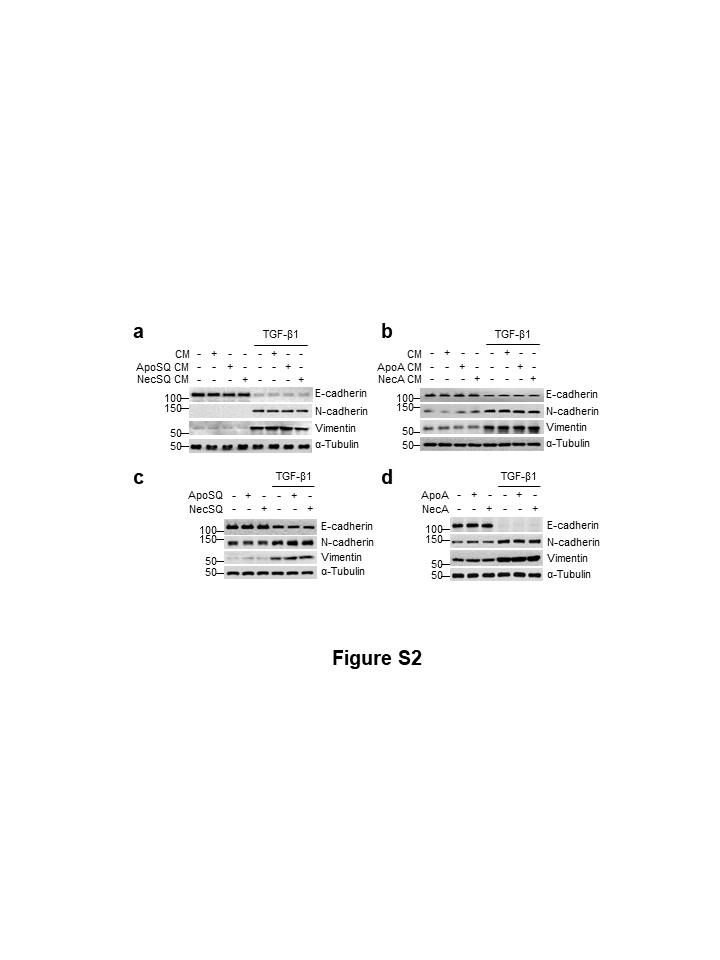


**Supplementary Fig. S4** Effect of direct exposure of cancer cells to corresponding apoptotic cancer cells on TGF-β1-induced EMT. **a** and **c** Immunoblot analysis of EMT markers (E-cadherin, N-cadherin, and vimentin) in 344SQ cell lysates. **b** and **d** Immunoblot analysis of EMT markers in A549 cell lysates. 344SQ (SQ) in **a** or A549 cells (A) in **b** were stimulated directly with ApoSQ, NecSQ, ApoA, or NecA for 24 h. Conditioned medium (CM) was added to the corresponding cancer cells in the absence or presence of TGF-β1 (10 ng/ml) for 48 h. SQ in **c** or A in **d** were stimulated directly with ApoSQ, NecSQ, ApoA, or NecA in the presence of TGF-β1 (10 ng/ml) for 48 h. Data are from one experiment representative of three independent experiments with similar results.

**
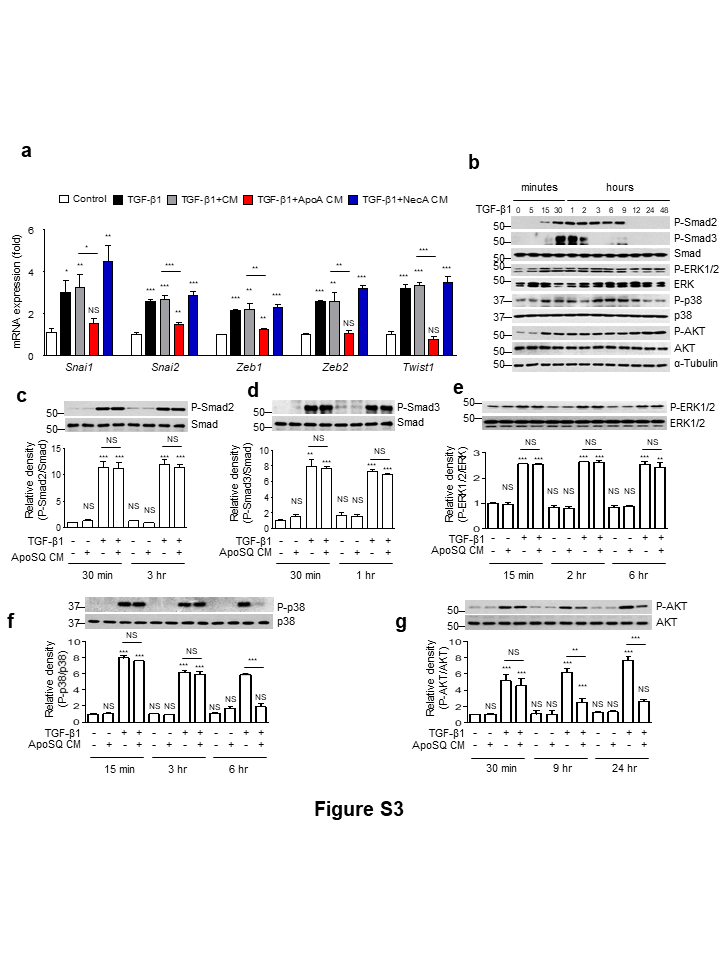
**

**Supplementary Fig. S5** Interaction between macrophages and apoptotic lung cancer cells inhibits TGF-β1 signaling in cancer cells. **a** Real-time PCR analysis of *Snai1*, *Snai2*, *Zeb1*, *Zeb2,* and *Twist1* mRNAs in A549 cell samples. A549 cells were treated with conditioned medium (CM) from RAW cells alone, or from RAW/apoptotic A549 cell (ApoA CM) or RAW/necrotic A549 cell (NecA CM) co-cultures in the presence of TGF-β1 (10 ng/ml) for 48 h. **b** Immunoblot analysis of phosphorylated Smad2, phosphorylated Smad3, total Smad, phosphorylated ERK1/2, total ERK, phosphorylated p38 MAP kinase, total p38 MAP kinase, Akt phosphorylated at S473, total Akt, and α-tubulin in 344SQ cell lysates. 344SQ cells were stimulated with TGF-β1 (10 ng/ml) for the indicated times. **c-g** Immunoblot analysis of phosphorylated Smad2 and total Smad, phosphorylated Smad3 and total Smad, phosphorylated ERK1/2 and total ERK1/2, phosphorylated p38 MAP kinase and total p38 MAP kinase, and phosphorylated Akt and total Akt in 344SQ cell lysates. *lower panel* the relative densitometric intensity of the indicated proteins. 344SQ cells were treated with CM from RAW/apoptotic 344SQ cell (ApoSQ CM) co-cultures in the presence of TGF-β1 (10 ng/ml) for the indicated times. NS: not significant; **P* < 0.05, ***P* < 0.01 and ****P* < 0.001. Data are from three independent experiments (mean ± s.e.m. in **a, c–g** *lower panel*), or from one experiment representative of three independent experiments with similar results (**b, c–g** *upper panel*).


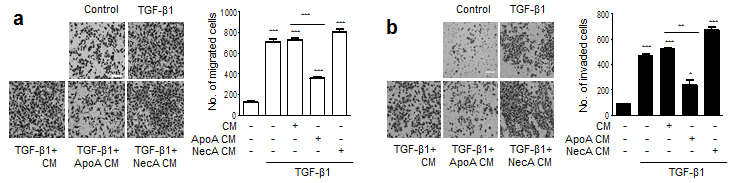


**Supplementary Fig. S6** Interaction between macrophages and apoptotic lung cancer cells inhibits migration and invasion of lung cancer cells. **a** Phase-contrast microscopy (*left*) and quantification of migrated A549 cells (*right)* for the analysis of migratory abilities using Fn-coated Transwell. **b** Phase-contrast microscopy (*left*) and quantification of invaded A549 cells (*right)* for the analysis of invasive abilities using Matrigel-coated Transwell plates. Scale bars: 100 μm in **a** and **b**. A549 cells were treated with conditioned medium (CM) from RAW cells alone, or from RAW/apoptotic A549 (ApoA CM) or RAW/necrotic A549 (NecA CM) cell co-cultures, in the presence of TGF-β1 (10 ng/ml) for 48 h. **P* < 0.05, ***P* < 0.01 and ****P* < 0.001. Data are from one experiment representative of three independent experiments with similar results (in **a** and **b** *left*, from three fields from replicate wells (mean ± s.e.m. in **a** and **b** *right*).


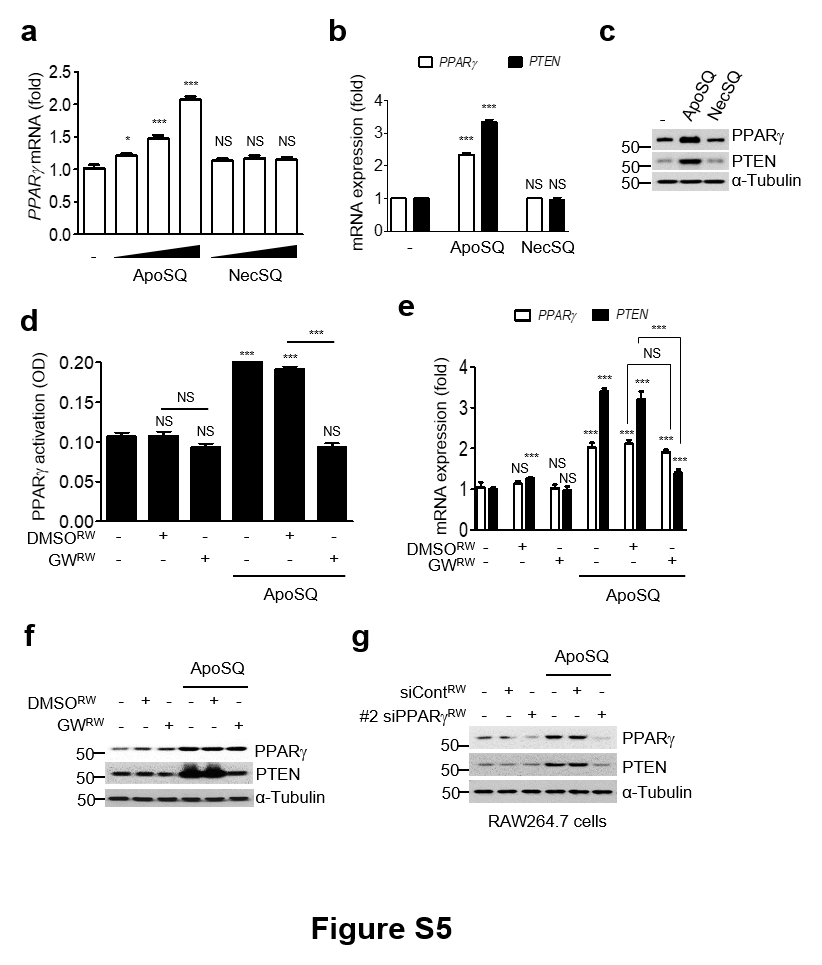


**Supplementary Fig. S7** Apoptotic 344SQ cells induces PPARγ-dependent PTEN expression in macrophages. **a** Real-time PCR analysis of *PPARγ* mRNAs in RAW cells stimulated with apoptotic (ApoSQ; 1, 2, or 3 × 10^5^/ml) or necrotic (NecSQ; 1, 2, or 3 × 10^5^/ml) 344SQ cells for 2 h. **b** Real-time PCR analysis of *PPARγ* and *PTEN* mRNAs in RAW cells stimulated with ApoSQ cells (3 × 10^5^/ml) or NecSQ cells (3 × 10^5^/ml) for 2 h for PPARγ mRNA analysis or for 24 h for PTEN mRNA analysis. **c** Immunoblot analysis of indicated proteins in RAW cells stimulated with ApoSQ (3 × 10^5^/ml) or NecSQ (3 × 10^5^/ml) for 24 h. **d** PPARγ activity in nuclear extracts of RAW cells pretreated with GW9662 (10 μM) for 1 h before stimulation with ApoSQ for 24 h. **e** Real-time PCR analysis of PPARγ and PTEN mRNAs in RAW cells pretreated with GW9662 (10 μM) for 1 h before stimulation with ApoSQ. **f** Immunoblot analysis of PPARγ and PTEN protein in RAW cells pretreated with GW9662 (10 μM) for 1 h before stimulation with ApoSQ for 24 h. **g** Immunoblot analysis of PPARγ and PTEN protein in RAW cells transfected with siRNA against PPARγ (#2 siPPARγ) for 24 h before stimulation with ApoSQ for 24 h. NS: not significant; **P* < 0.05 and ****P* < 0.001. Data are from three independent experiments (mean ± s.e.m. in **a, b, d** and **e**), or from one experiment representative of three independent experiments in **c, f** and **g**.


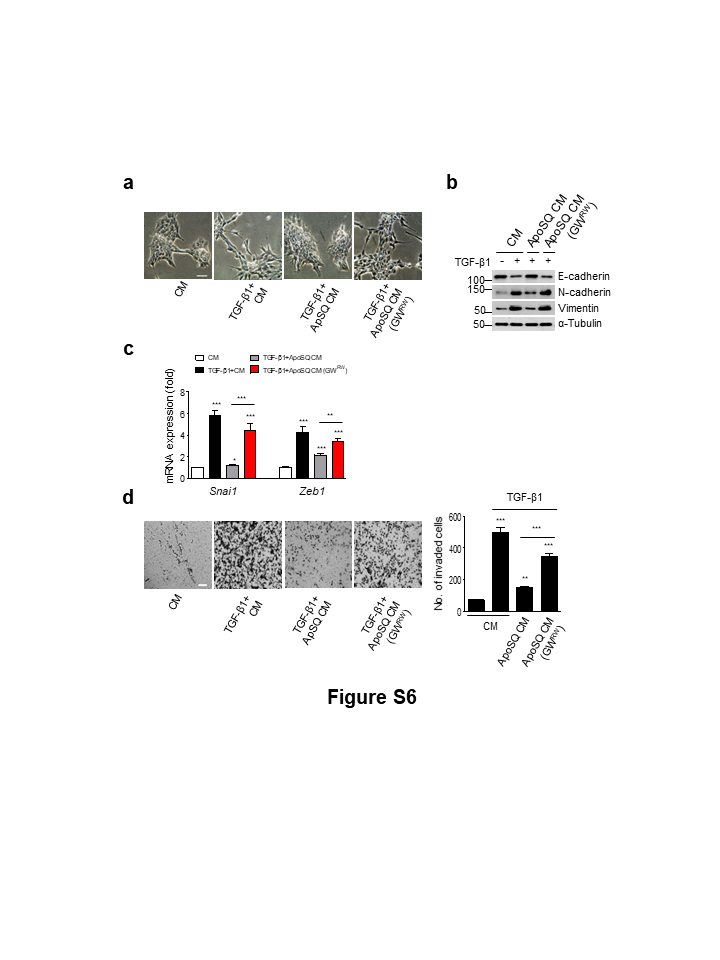


**Supplementary Fig. S8** Inhibition of PPARγ activity in macrophages reverses condition medium-induced anti-EMT and anti-invasive effects in 344SQ cells. **a** Morphological changes in the cells examined by phase-contrast microscopy. **b** Immunoblot analysis of indicated proteins in 344SQ cell lysates. **c** Real-time PCR analysis of *Snai1* and *Zeb1* mRNAs in 344SQ cells. **d** Phase-contrast microscopy (*left*) and quantification of invaded A549 cells (*right)* for the analysis of invasive abilities using Matrigel-coated Transwell plates. Scale bars: 100 μm in **a** and **d**. **a-d** RAW cells were pretreated with GW9662 (10 μM) for 1 h before stimulation with ApoSQ cells (3 × 10^5^/ml) for 24 h. Conditioned medium (CM) was added to 344SQ cells in the presence of TGF-β1 (10 ng/ml) for 48 h. NS: not significant; **P* < 0.05, ***P* < 0.01 and ****P* < 0.001. Data are from one experiment representative of three independent experiments in **a, b** and **d** *left*, three independent experiments (mean ± s.e.m in **c**), or from three fields from replicate wells (mean ± s.e.m in **d** *right*).


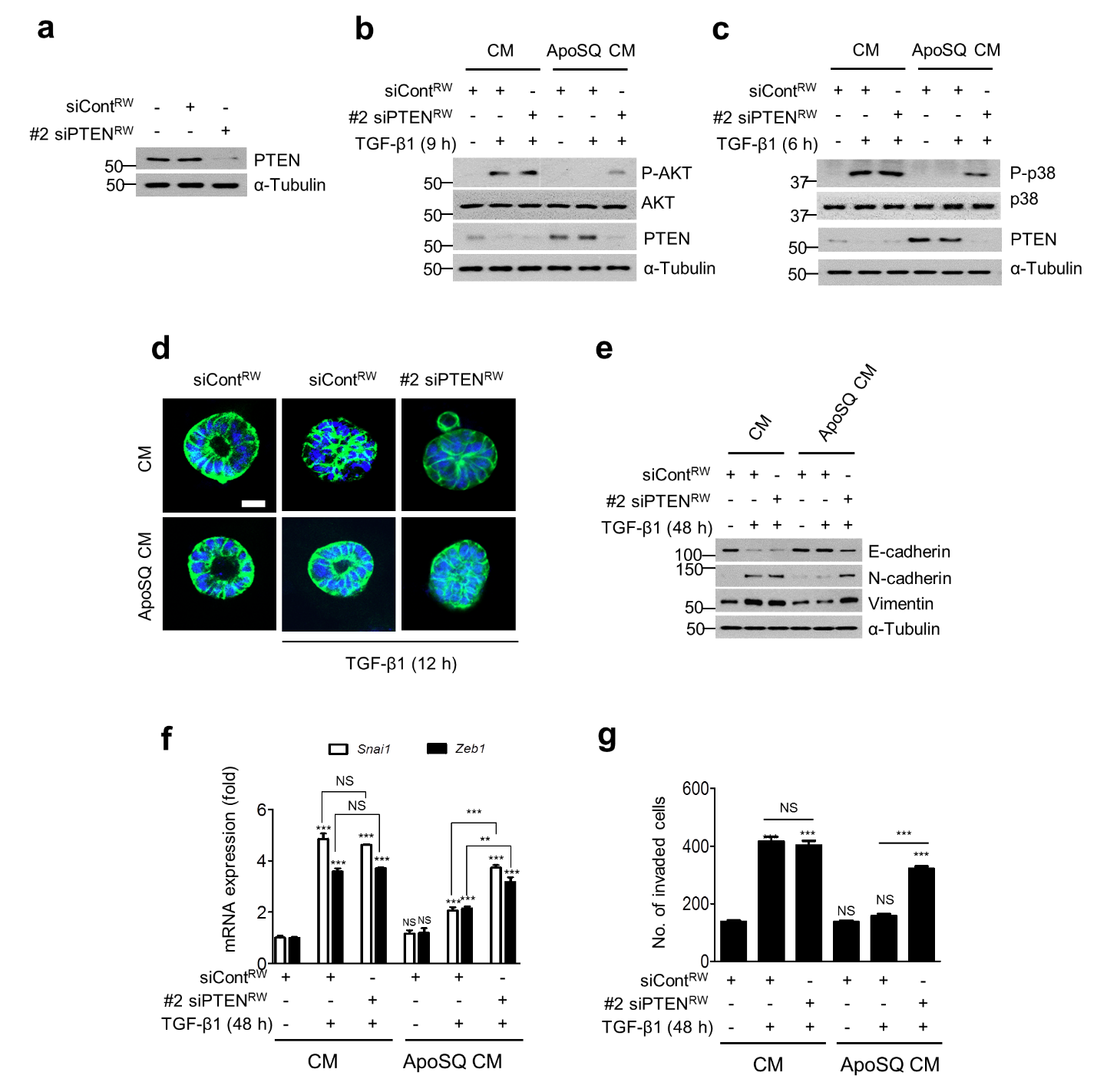


**Supplementary Fig. S9** PTEN Knockdown in macrophages reverses effects of ApoSQ CM on signaling, polarity, and EMT and invasion of 344SQ cells. **a** Immunoblot analysis of PTEN in RAW cells. RAW cells were transfected with siRNA of PTEN (#2 siPTEN) before apoptotic 344SQ (ApoSQ) stimulation for 24 h. **b** and **c** Immunoblot analysis of phosphorylated Akt and total Akt, and phosphorylated p38 MAP kinase and total p38 MAP kinase in 344SQ cell lysates. **d** Confocal microscope images of 3-D acini were taken 10 days after cell seeding. Normal acini of 344SQ cells were grown in 3D Matrigel containing CM and stained with anti-β-catenin (green) and DAPI. Scale bars: 20 μm. **e** Immunoblot analysis of EMT markers in 344SQ cell lysates. **f** qPCR analysis of *Snai1* and *Zeb1* mRNAs in 344SQ cells. **g** The invaded cell numbers were analyze their invasive ability using Matrigel-coated Transwell. **b-g** RAW cells were transfected with siRNA of PTEN (#2 siPTEN) before ApoSQ cell stimulation for 24 h. Conditioned medium (CM) was added to 344SQ cells with or without TGF-β1 (10 ng/ml) for the indicated time. NS, not significant; ****P* < 0.001. Data are from one experiment representative of three independent experiments with similar results in **a-e,** or three independent experiments (mean ± s.e.m. in **f** and **g**).


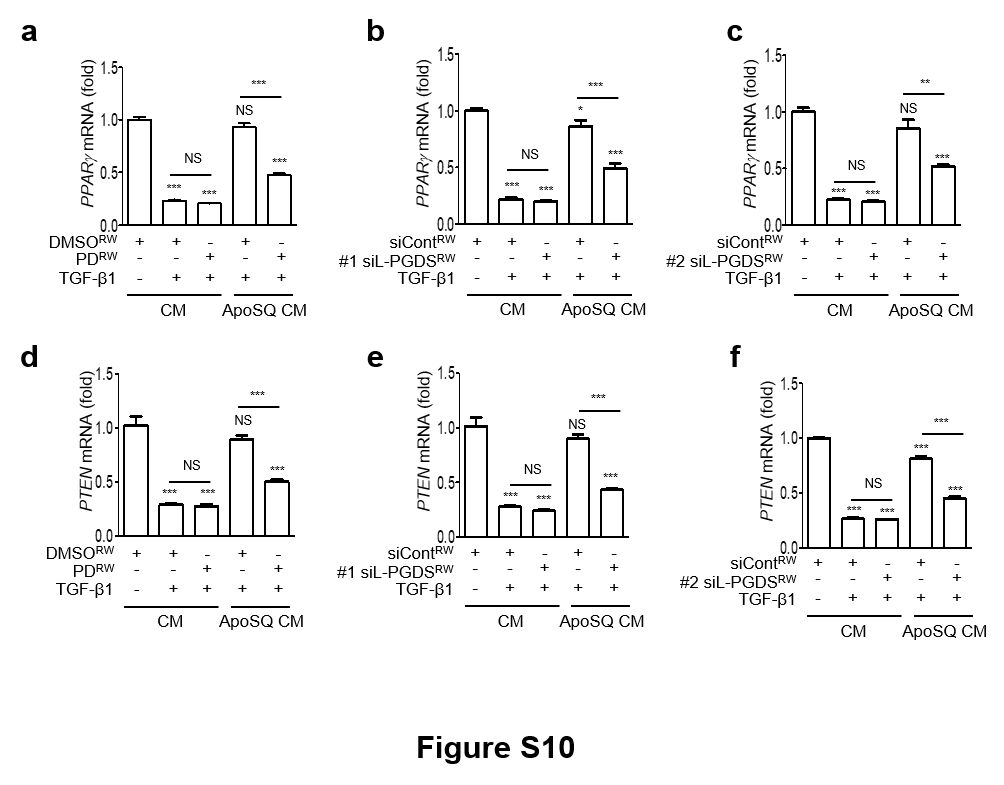


**Supplementary Fig. S10** Interaction of macrophages and apoptotic lung cancer cells inhibits ligand-dependent PPARγ/PTEN signaling. **a** Real-time PCR analysis of *PPARγ* mRNAs in 344SQ cell samples. RAW cells were pretreated with PD146176 for 1 h before stimulation with apoptotic 344SQ (ApoSQ) for 24 h. Conditioned medium (CM) was added to 344SQ cells in the presence of TGF-β1 (10 ng/ml) for 48 h. **b** and **c** Real-time PCR analysis of *PPARγ* mRNAs in 344SQ cell samples. RAW cells were transfected with two siRNAs against lipocalin-type prostaglandin D synthase (#1 and #2 siL-PGDS, respectively)**,** before stimulation with ApoSQ for 24 h. CM was added to 344SQ cells in the presence of TGF-β1 (10 ng/ml) for 48 h. **d** Real-time PCR analysis of *PTEN* mRNAs in 344SQ cell samples. RAW cells were pretreated with PD146176 for 1 h. CM was added to 344SQ cells in the presence of TGF-β1 (10 ng/ml) for 48 h. **e** and **f** Real-time PCR analysis of *PTEN* mRNAs in 344SQ cell samples. RAW cells were transfected with two siRNAs against #1 and #2 siL-PGDS, respectively, for 24 h before stimulation with ApoSQ cells for 24 h. CM was added to 344SQ cells in the presence of TGF-β1 (10 ng/ml) for 48 h. NS: not significant; **P* < 0.05, ***P* < 0.01 and ****P* < 0.001. Data are from three independent experiments (mean ± s.e.m in **a-f**).


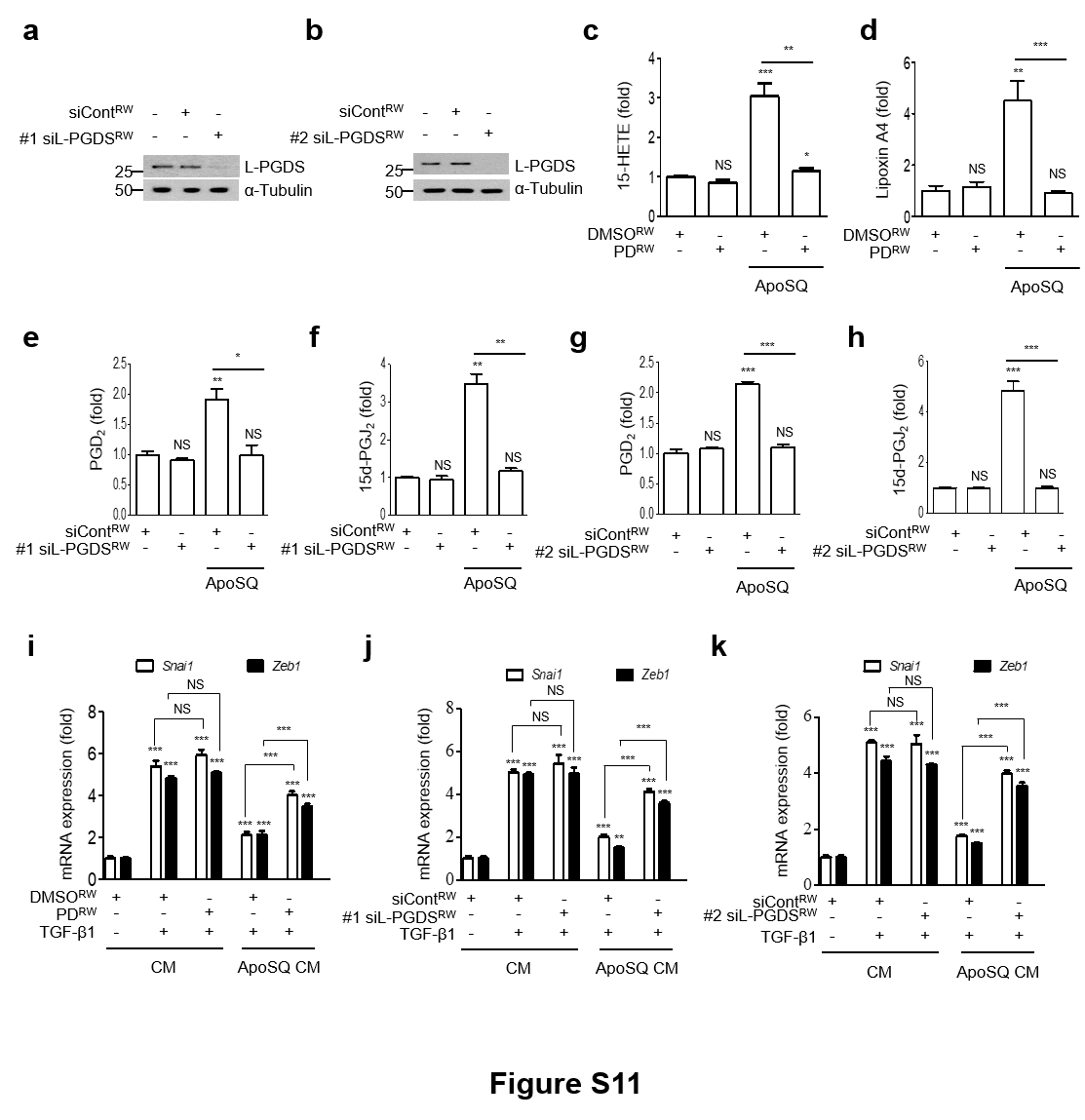


**Supplementary Fig. S11** Interaction of macrophages and apoptotic lung cancer cells inhibits EMT regulating transcription factor via PPARγ ligand-dependent signaling. **a** and **b** Immunoblot analysis of lipocalin-type prostaglandin D synthase (L-PGDS) expression in RAW cells transfected with two siRNAs against #1 and #2 siL-PGDS, respectively, for 24 h before stimulation with ApoSQ cells for 24 h. **c** and **d** ELISA of 15-HETE and lipoxin A4 in the condition medium (CM) from RAW cells pretreated with PD146176 1 h before stimulation with ApoSQ cells for 24 h. **e** and **g** ELISA of PGD_2_ in the CM from RAW cells transfected with two siRNAs against #1 and #2 siL-PGDS, respectively, for 24 h before stimulation with ApoSQ cells for 24 h. **f** and **h** ELISA of 15d-PGJ_2_ in the CM from RAW cells transfected with two siRNAs against #1 and #2 siL-PGDS, respectively, for 24 h before stimulation with ApoSQ cells for 24 h. **i-k** Real-time PCR analysis of *Snai1* and *Zeb1* mRNAs in 344SQ cell samples. **i** RAW cells were pretreated with PD146176 for 1 h. CM was added to 344SQ cells in the presence of TGF-β1 (10 ng/ml) for 48 h. **j** and **k** RAW cells were transfected with two siRNAs against #1 and #2 siL-PGDS, respectively, for 24 h before stimulation with ApoSQ cells for 24 h. CM was added to 344SQ cells in the presence of TGF-β1 (10 ng/ml) for 48 h. NS: not significant; **P* < 0.05, ***P* < 0.01 and ****P* < 0.001. Data are from one experiment representative of three independent experiments in **a** and **b**, or three independent experiments (mean ± s.e.m in **c-k**).

**
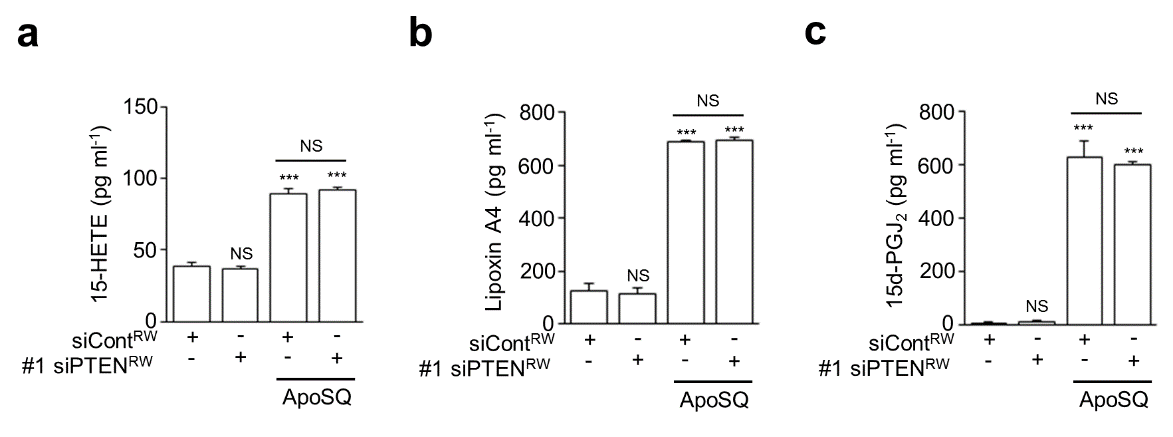
**

**Supplementary Fig. 12** PTEN Knockdown in macrophages does not inhibit production of PPARγ ligands by apoptotic 344SQ cells. **a-c** ELISA of 15-HETE, lipoxin A4 and PGJ_2_ in the CM from RAW cells transfected with #1 siRNA against PTEN for 24 h before stimulation with apoptotic 344SQ (ApoSQ) for 24 h. NS, not significant; ****P* < 0.001. Data are from three independent experiments (mean ± s.e.m. in **a**-**c**).


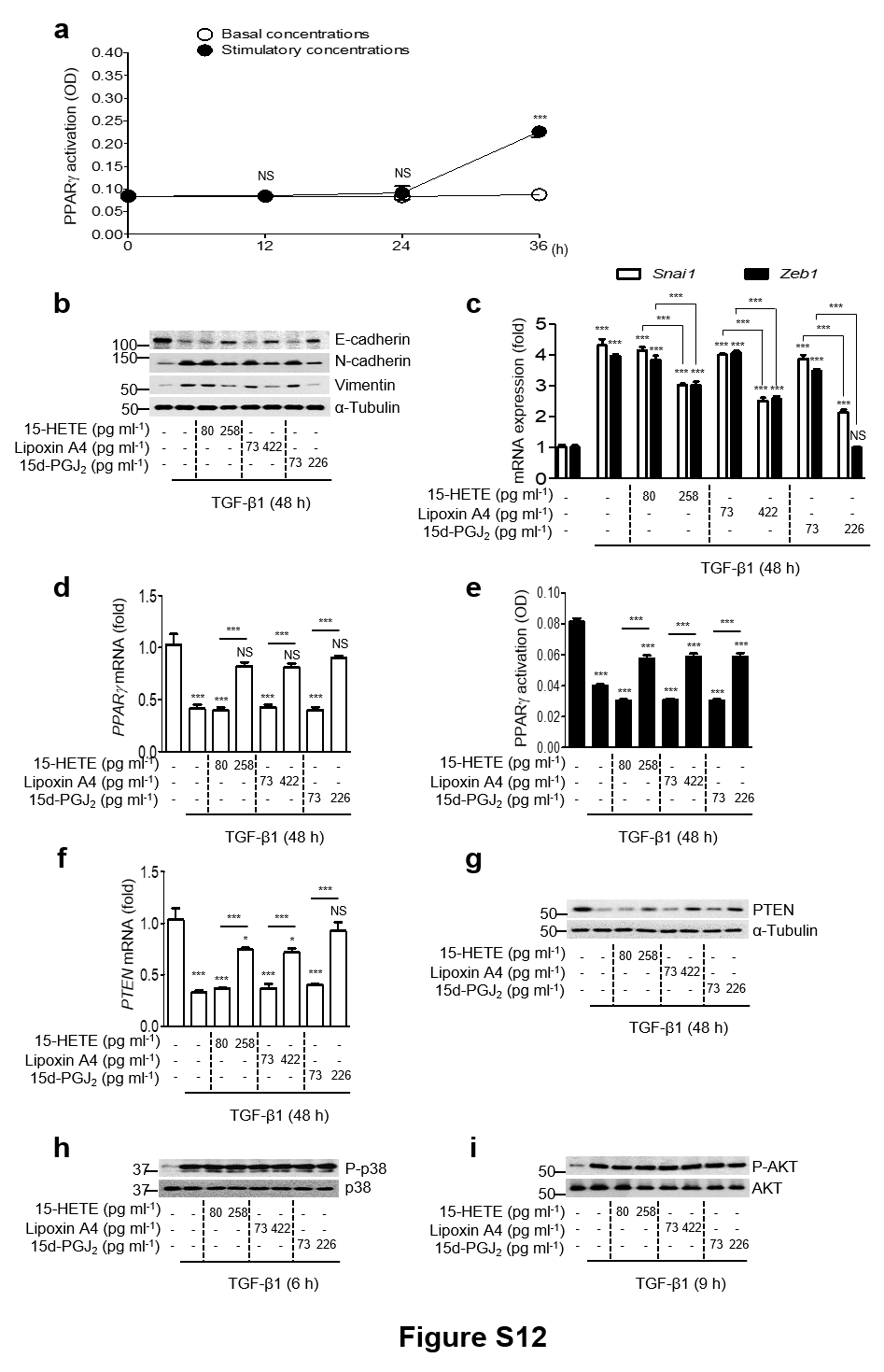


**Supplementary Fig. S13** Effects of exogenous 15-HETE, lipoxin A4, and 15d-PGJ_2_ on EMT, PPARγ/PTEN signaling, and signaling. **a** Time course of PPARγ activation in 344SQ cells treated with PPARγ ligands, such as15-HETE (80 and 258 pg/ml), lipoxin A4 (73 and 422 pg/ml), and 15d-PGJ_2_ (73 and 226 pg/ml), all together for the indicated times. **b** Immunoblot analysis of EMT markers in 344SQ cells treated individually with PPARγ ligands in the presence of TGF-β1 (10 ng/ml) for 48 h. **c** Real-time PCR analysis of *Snai1*, *Zeb1* in 344SQ cells treated individually with PPARγ ligands in the presence of TGF-β1 (10 ng/ml) for 48 h. **d** Real-time PCR analysis of *PPARγ* in 344SQ cells treated individually with PPARγ ligands in the presence of TGF-β1 (10 ng/ml) for 48 h. **e** PPARγ activation in nuclear extracts from 344SQ cells treated individually with PPARγ ligands in the presence of TGF-β1 (10 ng/ml) for 48 h. **f** Real-time PCR analysis of *PTEN* in 344SQ cells treated individually with PPARγ ligands in the presence of TGF-β1 (10 ng/ml) for 48 h. **g** Immunoblot analysis of PTEN in 344SQ cells treated individually with PPARγ ligands in the presence of TGF-β1 (10 ng/ml) for 48 h. **h** and **I** Immunoblot analysis of phosphorylated-p38 MAP kinase and total p38 MAP kinase, and phosphorylated Akt and total Akt in 344SQ cells treated individually with PPARγ ligands in the presence of TGF-β1 (10 ng/ml) for 6 and 9 h, respectively. NS: not significant; **P* < 0.05 and ****P* < 0.001. Data are from three independent experiments (mean ± s.e.m. in **a and c–f**), or from one experiment representative of three independent experiments in **b and g–i**.

**
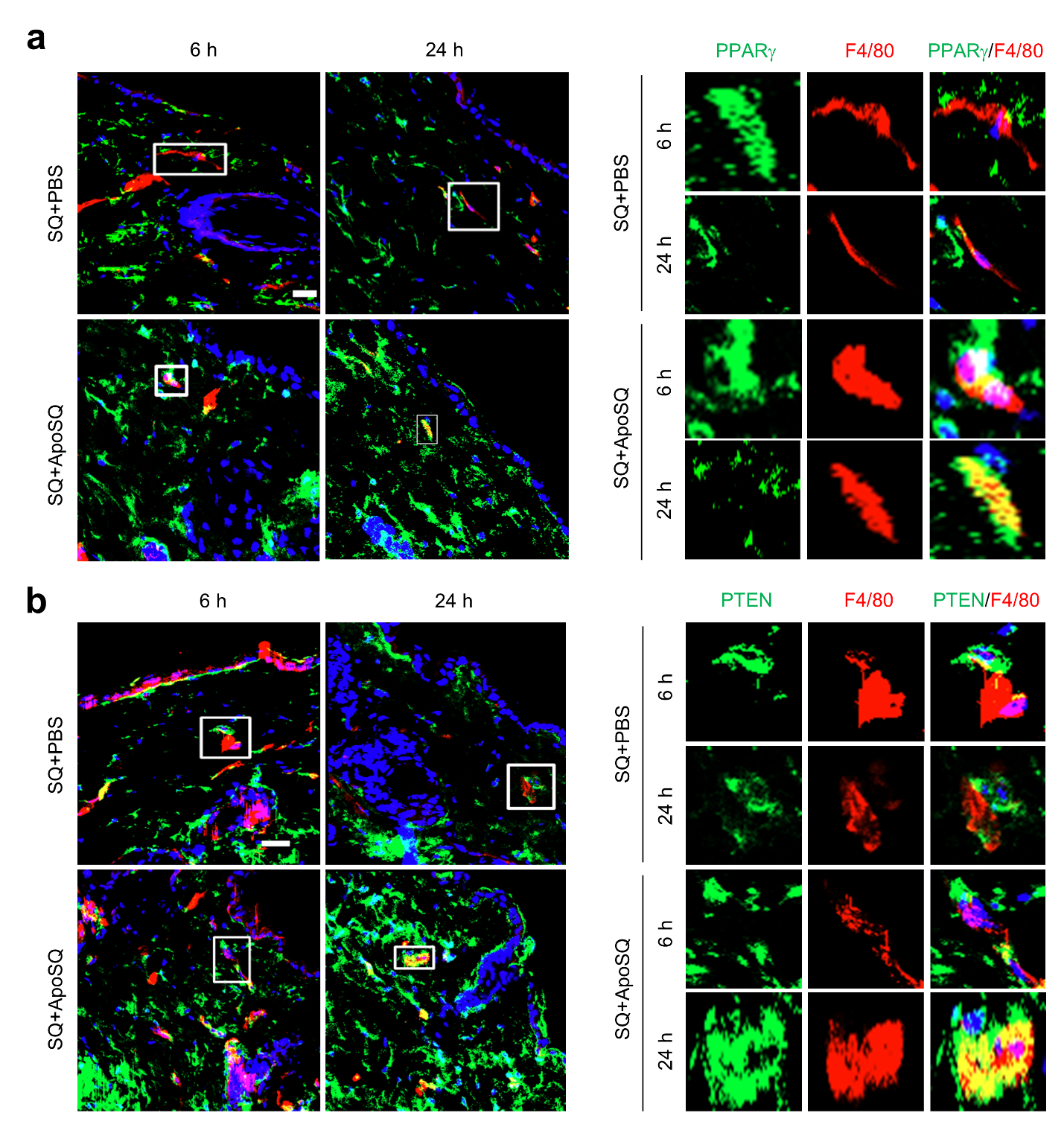
**

**Supplementary Fig. S14** PPARγ and PTEN induction in subcutaneous macrophages following apoptotic 344SQ cell injection. Phosphate-buffered saline (PBS) or apoptotic 344SQ cells (ApoSQ) were subcutaneously injected in the skin lesion 2 days after subcutaneous injection of 344SQ cells into wild-type B6129SF2/J mice. Mice were necropsied 6 or 24 h later. **a** Confocal images of macrophages expressing PPARγ in skin lesions immunostained with anti-F4/80 (red) or anti-PPARγ (green). **b** Confocal images of macrophages expressing PTEN in skin lesions immunostained with anti-F4/80 (red) or anti-PTEN (green). Full-size images of skin lesions in **a** and **b** *left panels* and enlarged ROIs from white squares on left panels in **a** and **b** *right panels*. Data are representative images from three mice per group.

**
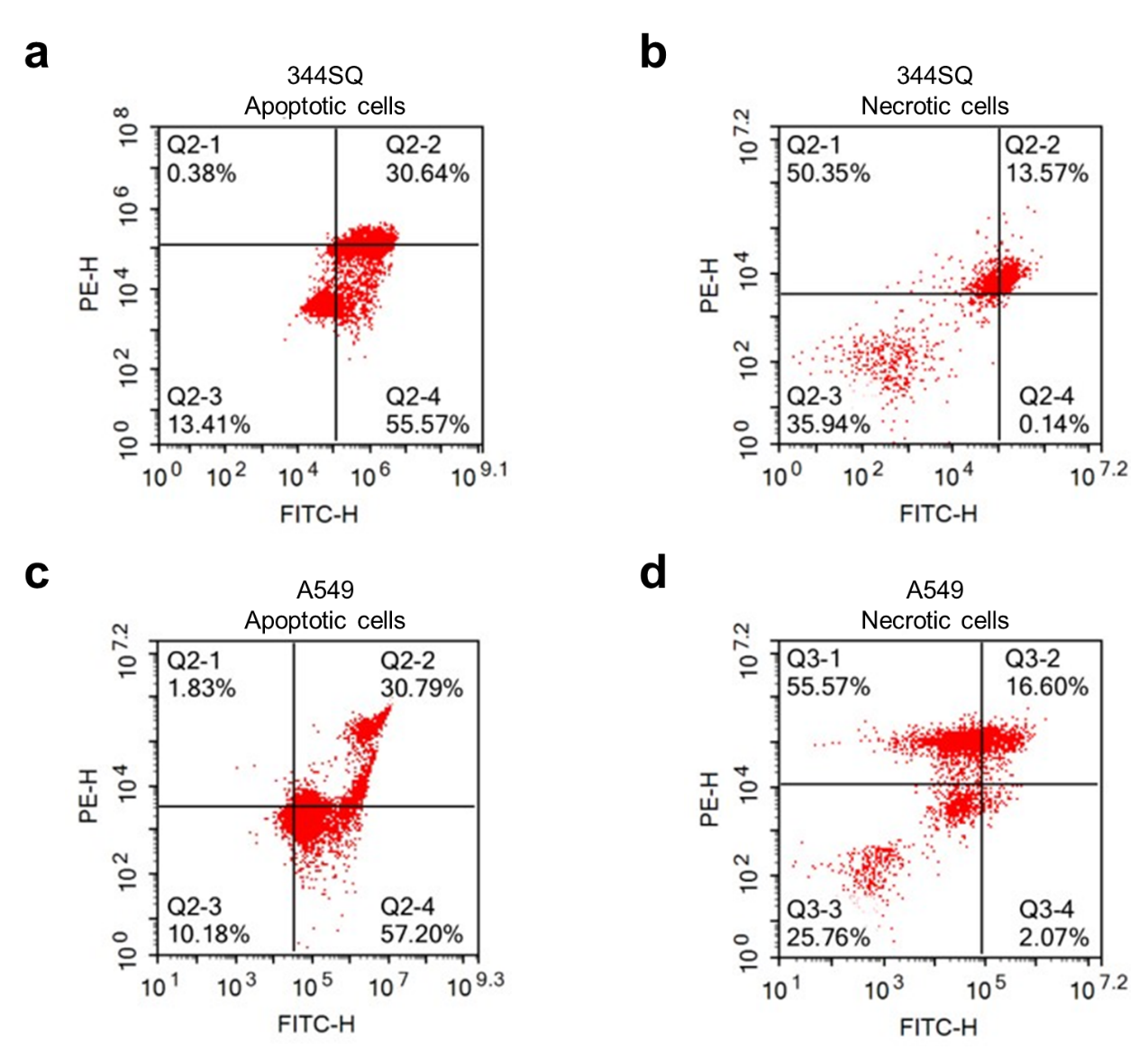
**

**Supplementary Fig. S15** Representative dot plots depicting percentage of 344SQ and A549 cells. Numbers within quadrants represent the percentage of cells within each quadrant. **a** and **c** Early apoptotic cells (annexin positive and propidium iodide negative) appear in the lower right quadrant. Late apoptotic cells (positive both annexin and propidium iodide) appear in the upper right quadrant. 344SQ and A549 cells were UV irradiated for 10 min followed by incubation in RPMI-1640 with 10% FBS for 2 h at 37°C and 5% CO2. **b** and **d** Necrotic cells (propidium iodide positive) appear in the upper left quadrant. Lysed (necrotic) apoptotic cancer cells were obtained by multiple freeze-thaw cycles. **a-d** Cells were stained with annexin-V and propidium iodide to discriminate between apoptotic and necrotic cell death.

**Supplementary tables**

**Supplementary Table S1.** List of antibodies used this study.

**
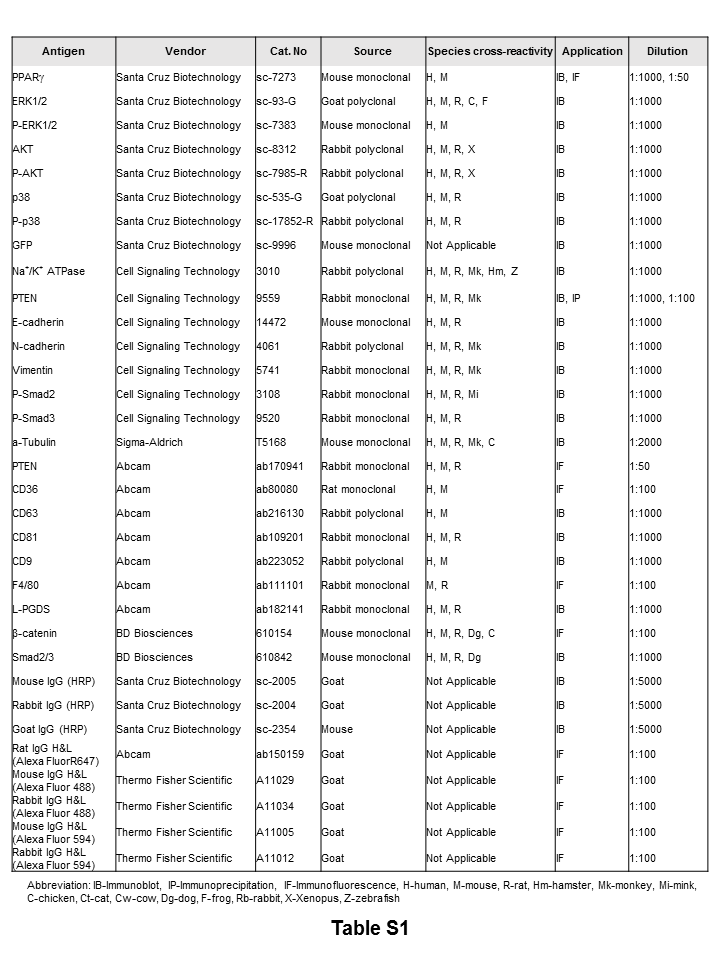
**

**Supplementary Table S2.** qPCR primers used in this study.


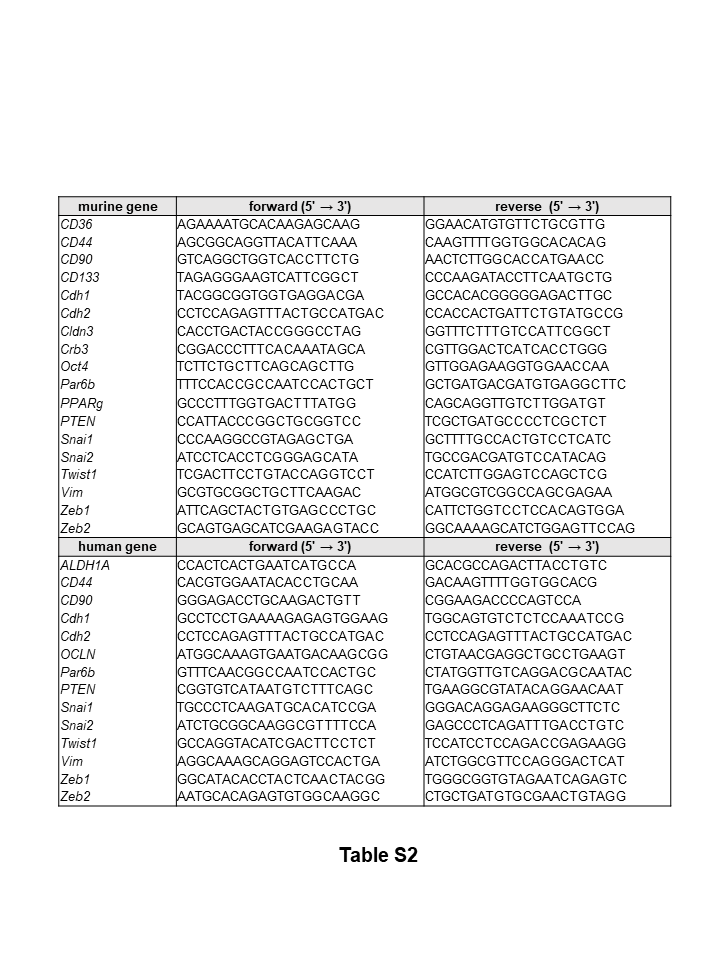

Supplement: Supplementary file 1 — Supplementary Materials [file 41423_2019_209_MOESM1_ESM.docx]
